# miR-142-5p and miR-130a-3p are regulated by IL-4 and IL-13 and control profibrogenic macrophage program

**DOI:** 10.1038/ncomms9523

**Published:** 2015-10-05

**Authors:** Shicheng Su, Qiyi Zhao, Chonghua He, Di Huang, Jiang Liu, Fei Chen, Jianing Chen, Jian-You Liao, Xiuying Cui, Yunjie Zeng, Herui Yao, Fengxi Su, Qiang Liu, Shanping Jiang, Erwei Song

**Affiliations:** 1 Guangdong Provincial Key Laboratory of Malignant Tumor Epigenetics and Gene Regulation, Medical Research Center, Sun Yat-Sen Memorial Hospital, Sun Yat-Sen University, Guangzhou 510120, China; 2 Breast Tumor Center, Sun Yat-Sen Memorial Hospital, Sun Yat-Sen University, Guangzhou 510630, China; 3 Department of Infectious Diseases, Third Affiliated Hospital, Sun Yat-Sen University, Guangzhou 510630, China; 4 Department of Pathology, Sun Yat-Sen Memorial Hospital, Sun Yat-Sen University, Guangzhou 510630, China; 5 Department of Oncology, Sun Yat-Sen Memorial Hospital, Sun Yat-Sen University, Guangzhou 510630, China; 6 Department of Respiratology, Sun Yat-Sen Memorial Hospital, Sun Yat-Sen University, Guangzhou 510120, China

## Abstract

Macrophages play a pivotal role in tissue fibrogenesis, which underlies the pathogenesis of many end-stage chronic inflammatory diseases. MicroRNAs are key regulators of immune cell functions, but their roles in macrophage's fibrogenesis have not been characterized. Here we show that IL-4 and IL-13 induce miR-142-5p and downregulate miR-130a-3p in macrophages; these changes sustain the profibrogenic effect of macrophages. *In vitro*, miR-142-5p mimic prolongs STAT6 phosphorylation by targeting its negative regulator, SOCS1. Blocking miR-130a relieves its inhibition of PPARγ, which coordinates STAT6 signalling. *In vivo*, inhibiting miR-142-5p and increasing miR-130a-3p expression with locked nucleic acid-modified oligonucleotides inhibits CCL_4_-induced liver fibrosis and bleomycin-induced lung fibrosis in mice. Furthermore, macrophages from the tissue samples of patients with liver cirrhosis and idiopathic pulmonary fibrosis display increased miR-142-5p and decreased miR-130a-3p expression. Therefore, miR-142-5p and miR-130a-3p regulate macrophage profibrogenic gene expression in chronic inflammation.

## Abstract (web summary)

Fibroblast activity is regulated by tissue macrophages. Here the authors show that two miRNAs regulated by IL-4 and IL-13 in macrophages target SOCS1 and PPARγ and modulate profibrogenic macrophage program *in vitro* and *in vivo*, and that alterations of these miRNAs are found in fibrosis.

## Graphical abstract

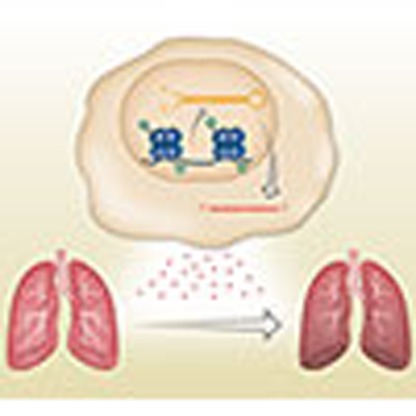

Fibrosis is responsible for progressive organ failure in many chronic inflammatory diseases. Macrophages (Mϕ) are key drivers of fibrogenesis and found in close proximity with collagen-producing myofibroblasts^1^. Classically activated (M1) macrophages induced by Toll-like receptor and T helper type 1 (Th1) signals and alternatively activated (M2) macrophages activated by Th2 signals are the two extremes of the spectrum of functional macrophage states^2^,^3^. Th2 cytokines, such as IL-4 and IL-13, are abundant in chronic infectious and autoimmune diseases^4^,^5^, and contribute to extensive tissue fibrosis in mouse models^6^. IL-4/IL-13-induced macrophages activate fibroblasts by secreting profibrogenic cytokines. Blocking TGF-β secretion from macrophages inhibits liver fibrosis in mouse models^1^. CCL18, another cytokine induced by IL-4/IL-13 (ref. 7), is secreted by lung macrophages in idiopathic pulmonary fibrosis (IPF) patients and induces collagen production by fibroblasts^8^,^9^.

Macrophage polarization is controlled by various transcriptional factors. STAT1 is responsible for M1 polarization, whereas STAT6 mediates M2 activation^10^. Although IL-4/IL-13 activates the STAT6 pathway for M2 polarization, these cytokines induce negative feedback to inhibit STAT6 phosphorylation by upregulating suppressor of cytokine signalling 1 (SOCS1), which competes with STAT6 for phosphorylation binding sites of JAK^2^. Therefore, M2 macrophages require additional mechanisms that overcome the elevated SOCS1 to maintain sufficient STAT6 phosphorylation and their perpetuation of fibrogenesis in chronic inflammatory diseases. In addition, peroxisome proliferator-activated receptor γ (PPARγ), which is upregulated by IL-4/IL-13 and synergistically interacts with STAT6 at target gene promoters, is also necessary for M2 activation^2^. A protein synthesis inhibitor could not abrogate IL-4-augmented PPARγ expression, suggesting that IL-4-mediated PPARγ upregulation does not require the synthesis of a new protein^11^. Nevertheless, the mechanisms by which IL4/IL-13 increase PPARγ expression have not been fully explored.

Emerging evidence has shown that post-transcriptional regulation by microRNAs (miRNAs) may coordinate transcriptional factors to determine cell fates^12^. A panel of miRNAs are induced by LPS and participate in the proinflammatory response of M1 macrophages^12^,^13^,^14^,^15^,^16^,^17^. Despite the roles of miRNAs documented in M1 activation, whether miRNAs also contribute to the polarization of IL-4/IL-13-activated macrophages and their profibrogenesis remains largely unknown. Here, we report that increased miR-142-5p and decreased miR-130a-3p induced by IL4/IL13 target SOCS1 and PPARγ, respectively, and regulate the profibrogenesis of macrophages in chronic inflammation.

## Results

### IL-4/IL-13 alter the miRNA expression profiles in Mϕ

To evaluate the profibrogenic capability of various macrophage subsets, we treated primary human monocyte-derived macrophages with a panel of macrophage-activating cytokines, and examined their capability to activate primary human fibroblasts upon co-culturing. We observed that IL-4- and IL-13-activated macrophages (M(IL-4) and M(IL-13), respectively) strongly promoted collagen contraction and proliferation of fibroblasts, while M(IL-10) and M(TGF-β) were much weaker (Supplementary Fig. 1a–c). In contrast, M(LPS) and M(IFN-γ) did not promote but instead inhibited fibroblast activation, which was consistent with previous reports^18^. These data indicated that IL-4/IL-13-activated macrophages are the highly profibrogenic macrophage subsets.

**Figure 1:**
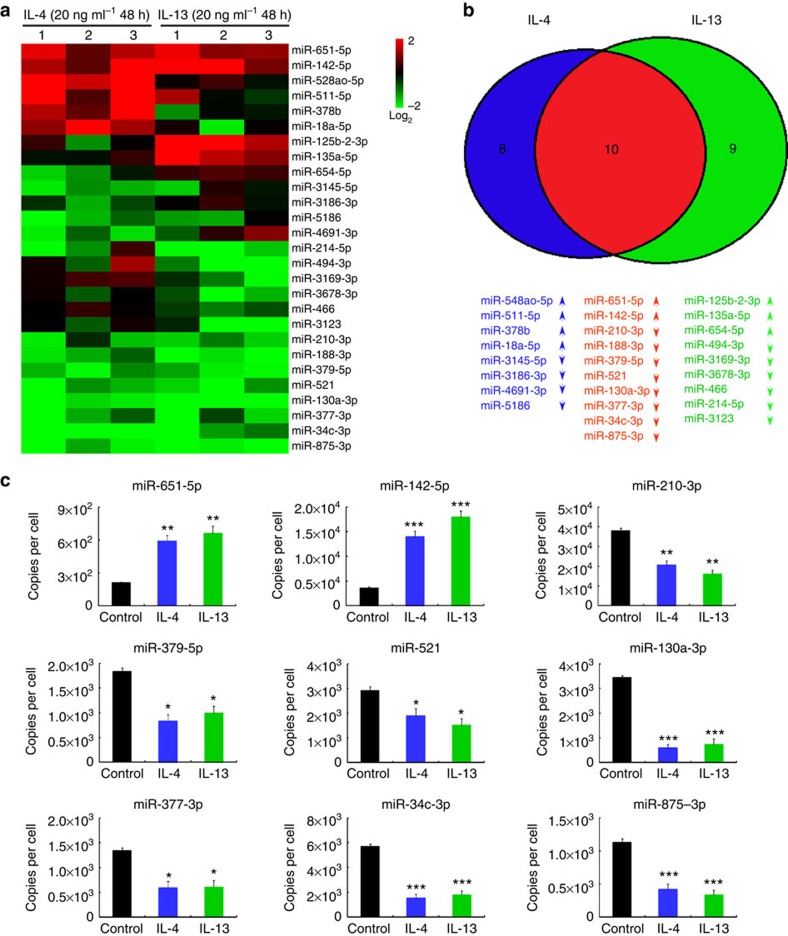
IL-4/IL-13 alter the miRNA expression profiles in macrophages. (**a**) Primary human macrophages derived from peripheral blood monocytes were treated for 48 h with 20 ng ml^−1^ IL-4 or IL-13, and the miRNA expression profiles were analysed by microarray. The mean fluorescence intensity was calculated as the average for three replicates. The results are presented as the miRNA ratio versus the untreated cells (*n*=3 per group, fold change >2, *P*<0.05 by two way ANOVA). (**b**) Venn diagram of all miRNAs with different expression in the cells treated as in (**a**). Red indicates the overlap between IL-4-regulating miRNAs and IL-13-regulating mRNAs. (**c**) Absolute quantification of the expression of the indicated miRNAs at 48 h following IL-4/IL-13 treatment as determined by qRT–PCR (mean±s.e.m., *n*=4; *P<0.05; **P<0.01; ***P<0.001, compared with untreated macrophages by two-tailed Student's *t*-test). ANOVA, analysis of variance.

To examine the miRNA expression profiles of the profibrogenic macrophages, we compared miRNA expression in the untreated macrophages versus M(IL-4) and M(IL-13) using miRNA microarray. IL-4 and IL-13 treatments resulted in marked changes in the expression of 18 and 19 miRNAs in the macrophages, respectively (Fig. 1a). Among these miRNAs, the expression of 10 miRNAs was altered consistently in both M(IL-4) and M(IL-13) (Fig. 1b), and changes in 9 of these miRNAs could be validated by quantitative PCR with reverse transcription (qRT–PCR) (Fig. 1c and Supplementary Fig. 1d). Of note, maintenance of the changes in miRNA expression (Supplementary Fig. 1e) in the macrophages depends on the sustained presence of IL-4/IL-13.

### miR-142-5p and 130a-3p control M2 polarization

To determine the function of the above miRNAs in M2 polarization, we transduced macrophages with anti-sense oligonucleotides (ASO) for each upregulated miRNA or miRNA mimics for each downregulated miRNA using lentiviral vectors. Because CCL18 is a major chemokine in human M2-macrophages that promotes fibrogenesis^7^,^8^, we evaluated CCL18 secretion by the macrophages upon tranduction with miRNA mimics or ASOs. We observed that transduction of the macrophages with miR-142-5p ASO or miR-130a-3p mimics, but not with other oligonucleotides, reduced CCL18 secretion following IL-4/IL-13 treatments (Supplementary Fig. 2a).

**Figure 2:**
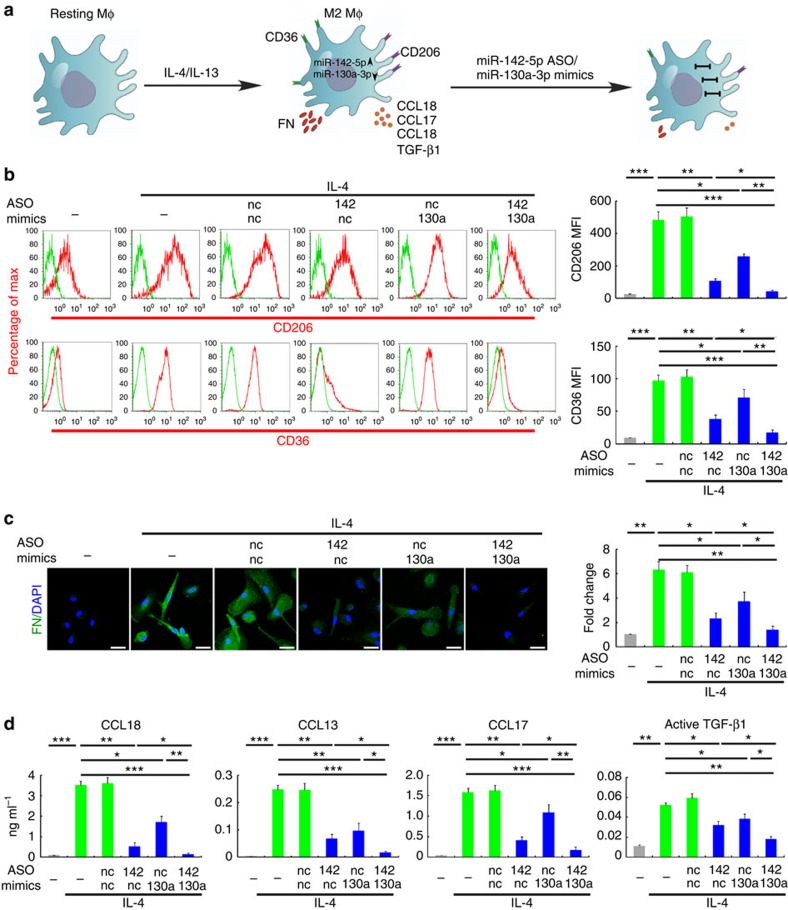
miR-142-5p and miR-130a-3p regulate M2 activation of macrophages. (**a**–**d**) Human macrophages were transduced with nc, miR-142-5p ASO or miR-130a-3p mimics or both using lentiviral vectors. After 24 h, the cells were treated with IL-4 for 48 h. (**a**) The schematics of the approach. (**b**) Expression of CD206 and CD36 in macrophages as determined by flow-cytometry analysis. The representative histograms and quantitation of the MFI are shown (mean±s.e.m., *n*=4 independent experiments; *P<0.05; **P<0.01; ***P<0.001. *P* values were obtained using two-tailed Student's *t*-test). (**c**) Representative images of fluorescent fibronectin(FN)/DAPI staining in macrophages. Scale bar, 20 μm. Fluorescence intensity was quantitated by ImageJ software and normalized to control levels (mean±s.e.m., *n*=3 independent experiments; *P<0.05; **P<0.01. *P* values were obtained using two-tailed Student's *t*-test). (**d**) Cytokine levels in the media of macrophages (mean±s.e.m., *n*=4 independent experiments; *P<0.05; **P<0.01; ***P<0.001. *P* values were obtained using a two-tailed Student's *t*-test). MFI, mean fluorescence intensity; nc, negative control.

To further confirm the roles of miR-142-5p and miR-130a-3p in M2 polarization, we transduced M(IL-4) with miR-142-5p ASO or/and miR-130a-3p mimics and adjusted their miRNA levels to those in the untreated cells (Supplementary Fig. 2b). Then, we examined the secretion of other M2 cytokines, including CCL17, CCL13 and TGF-β1; the expression of M2 surface markers, including CD206 and CD36, an M2 profibrogenic factor, fibronectin^19^; and pinocytosis of horseradish peroxidase which has been noted to increase in M2 macrophages^20^ (Fig. 2a). Transduction of macrophages with either miR-142-5p ASO or miR-130a-3p mimics significantly reduced the expression of M2 markers (Fig. 2b–d and Supplementary Fig. 2c,d) and pinocytosis (Supplementary Fig. 2e) induced by IL-4. Of note, upregulation of the M2 markers and pinocytosis induced by IL-4 was almost completely abrogated when both miR-142-5p ASO and miR-130a-3p mimics were transduced.

To determine whether the effects of blocking miR-142-5p and supplementing miR-130a-3p are synergistic, we employed a combination index (CI) to indicate synergistic (CI<1), additive (CI=1) and antagonistic (CI>1) effects of miR-142-5p ASO and miR-130a-3p mimics on M2 cytokine production, respectively. Transducing M(IL-4) with miR-142-5p ASO and miR-130a-3p mimics synergistically reduced M2 cytokine production as CI values were <1 in all combined assays (Supplementary Table 1). Similar results were observed in M(IL-13) (Supplementary Fig. 2f). Collectively, our data suggested that miR-142-5p and miR-130a-3p synergistically control M2 macrophage polarization.

### miR-142-5p and 130a-3p regulate Mϕ profibrogenesis

**Figure 3:**
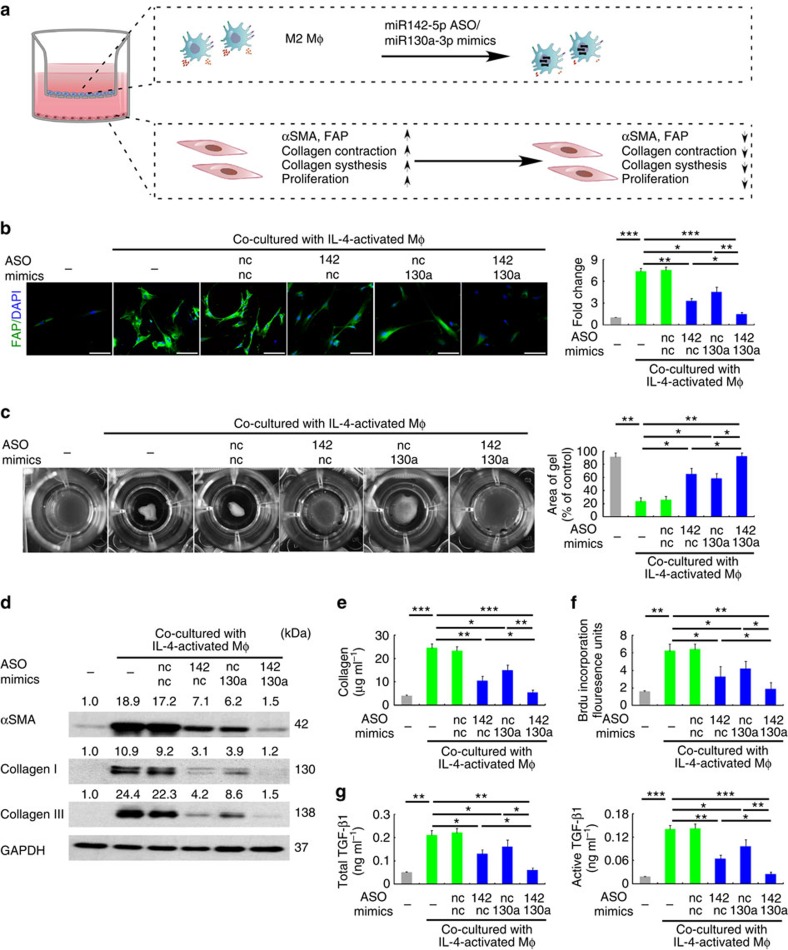
miR-142-5p and miR-130a-3p regulate the profibrogenesis of macrophages. (**a**–**g**) Human macrophages were transduced with control, miR-142-5p ASO or miR-130a-3p mimics or both. After 24 h, the macrophages were stimulated with IL-4 for 12 h and co-cultured with primary human fibroblasts for another 48 h. (**a**) The schematics of the approach. (**b**) Representative images of fluorescent FAP/DAPI staining in fibroblasts. Scale bar, 100μm. Fluorescence intensity was quantitated.(mean±s.e.m., *n*=3 independent experiments; *P<0.05; **P<0.01. *P* values were obtained using two-tailed Student's *t*-test). (**c**) Fibroblast contractility in three-dimensional collagen matrices (mean±s.e.m., *n*=4 independent experiments; *P<0.05; **P<0.01. *P* values were obtained using two-tailed Student's *t*-test). (**d**) Representative images of western blot analysis of α-SMA, collagen I and collagen III in fibroblasts (*n*=3). The numbers above the blots present the intensity ratio of indicated protein/GAPDH analysed by ImageJ. (**e**) Extracellular acid-soluble collagen production of fibroblasts was measured by the Sircol assay. (mean±s.e.m., *n*=4 independent experiments; *P<0.05; **P<0.01; ***P<0.001. *P* values were obtained using two-tailed Student's *t*-test). (**f**) The proliferation of fibroblasts was determined by BrdU incorporation assay (mean±s.e.m., *n*=4 independent experiments; *P<0.05; **P<0.01. *P* values were obtained using two-tailed Student's *t*-test). (**g**) Total TGF-β1 (acid-treated) and active TGF-β1 (not acid-treated) in the media of the macrophage/fibroblast co-culture system (mean±s.e.m., *n*=4 independent experiments; *P<0.05; **P<0.01; ***P<0.001. *P* values were obtained using two-tailed Student's *t*-test).

Because M(IL-4) promote fibrogenesis by activating fibroblasts^6^,^18^, we evaluated whether M(IL-4) induction by changes in miR-142-5p and miR-130a-3p expression may mediate the activation of co-cultured fibroblasts by examining their expression levels of α-smooth muscle actin (α-SMA) and fibroblast activation protein (FAP), which are features of fibroblast activation^21^,^22^ (Fig. 3a). Transduction of M(IL-4) with miR-142-5p ASO and miR-130a-3p mimics abrogated the ability of macrophages to induce FAP and α-SMA expression in the fibroblasts (Fig. 3b,d). Additionally, co-culturing the fibroblasts with M(IL-4) enhanced the ability of the fibroblasts to contract collagen gel; this enhancement was substantially attenuated when miR-142-5p was reduced or/and miR-130a-3p was increased in the co-cultured macrophages (Fig. 3c). Furthermore, transducing the M(IL-4) with miR-142-5p ASO and miR-130a-3p mimics synergistically inhibited their ability to stimulate collagen production and proliferation of fibroblasts when co-cultured (Fig. 3d–f and Supplementary Table 2). Additionally, TGF-β1 expression and activation in the co-cultured fibroblasts were suppressed by miR-142-5p and miR-130a-3p (Fig. 3g). Similar results were also obtained in M(IL-13) (Supplementary Fig. 3a–d). To investigate whether miR-142-5p and miR-130a-3p could directly mediate the TGF-β1 expression in fibroblasts, we examined their expression levels of these miRNAs in fibroblasts. Our data showed that their expression levels in fibroblasts were much lower than those in macrophages, particularly the levels of miR-142-5p (Supplementary Fig. 3e). Knockdown or forced expression of miR-142-5p or miR-130a-3p in fibroblasts had no appreciable effects of TGF-β1 expression or activation (Supplementary Fig. 3f).Collectively, our data suggested that miR-142-5p downregulation and miR-130a-3p upregulation synergistically enhance the profibrogenic activities of macrophages.

### miR-142-5p and 130a-3p target SOCS1 and PPARγ, respectively

**Figure 4:**
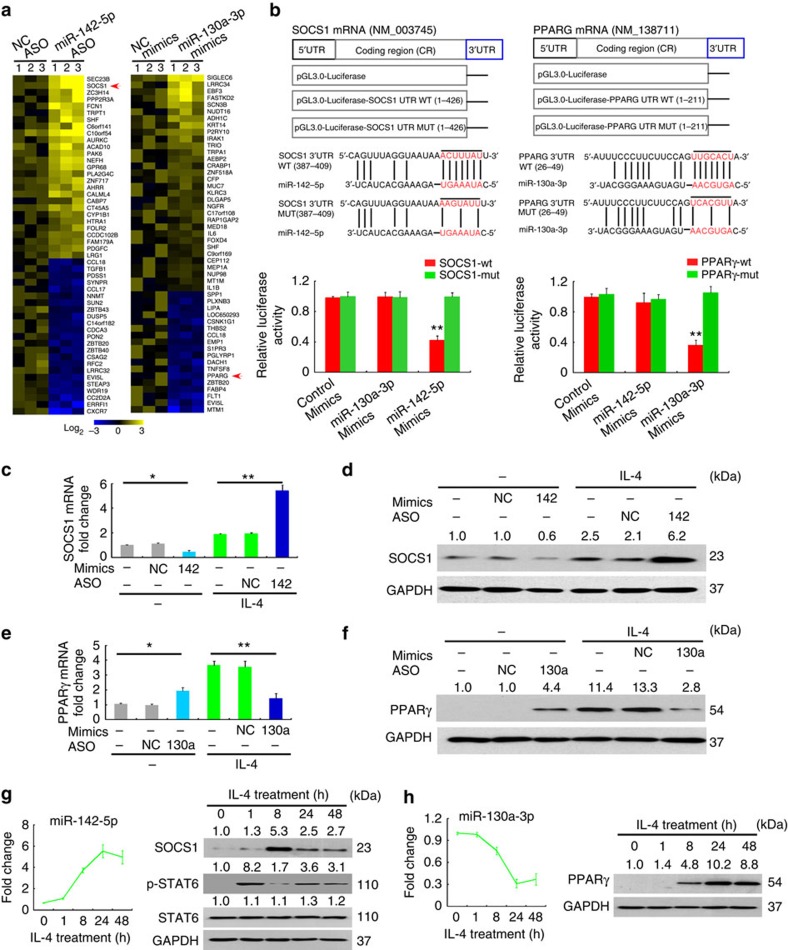
miR-142-5p and miR-130a-3p target the 3′UTRs of SOCS1 and PPAR**γ** respectively. (**a**) Heat map for gene-expression profiling of human M(IL-4) transduced with control ASO versus miR-142-5p ASO or control mimics versus miR-130-3p mimics. The mean fluorescence intensity was calculated as the average for three replicates, and the levels of expression were shown with log2-transformed values. The top 50 genes with the greatest changes compared with controls are shown (*n*=3). (**b**) Luciferase reporter assays for THP-1 cells transfected with pRL-TK vectors carrying SOCS1–3′ UTR versus SOCS1-mut-3′UTR or PPARγ-3′ UTR versus PPARγ-mut-3′ UTR in the absence or presence of the indicated miRNA mimics (mean±s.e.m., *n*=3 independent experiments; **P<0.01 compared to control by two-tailed Student's *t*-test). (**c**,**d**) The human untreated and M(IL-4) were transduced with miR-142-5p mimics and miR-142-5p ASO, respectively. Expression of SOCS1 (**c**) mRNA and (**d**) protein was measured. The numbers above the blots present the intensity ratio of SOCS1/GAPDH analysed by ImageJ. (mean±s.e.m., *n*=3, *P<0.05; **P<0.01 by two-tailed Student's *t*-test). (**e**,**f**) The human macrophages untreated and M(IL-4) were transduced with miR-130-3p ASO and miR-130-3p mimics, respectively. Expression of PPARγ (**e**) mRNA and (**f**) protein was measured (mean±s.e.m., *n*=3, *P<0.05; **P<0.01 by two-tailed Student's *t*-test). (**g**) Time course of miR-142-5p expression in human macrophages as determined by qRT–PCR and SOCS1 protein level and STAT6 phosphorylation as determined by western blotting after IL-4 stimulation (mean±s.e.m., *n*=3). (**h**) Time course of miR-130a-3p expression in human macrophages as determined by qRT–PCR and PPARγ protein level as determined by western blotting after IL-4 stimulation (mean±s.e.m., *n*=3).

To identify the target genes of miR-142-5p and miR-130a-3p that are involved in M2 polarization, we examined the mRNA expression profile changes after altering miR-142-5p or miR-130a-3p expression in M(IL-4). Of the most significantly differentially expressed genes in M(IL-4) when transduced with miR-142-5p ASO or miR-130a-3p mimics, two master regulators of M2 polarization^2^, SOCS1 expression was elevated and PPARγ expression was reduced (Fig. 4a), suggesting that these genes are potential targets of miR-142-5p and miR-130a-3p, respectively.

To validate these targets, we generated reporter constructs containing the 3′ UTR of SOCS1 (SOCS1-3′ UTR) and PPARγ (PPARγ-3′ UTR), respectively (Fig. 4b), and co-transfected them with the miRNA mimics. Co-transfection with miR-142-5p mimics specifically reduced the luciferase activity of the SOCS1-3′ UTR transfected cells, while miR-130a-3p mimics reduced that of the PPARγ-3′ UTR transfected cells (Fig. 4b). Furthermore, mutations in the miRNA seed sequences completely abrogated luciferase reduction induced by the miRNA mimics, confirming the specificity of the miRNA target sites (Fig. 4b). Therefore, SOCS1 and PPARγ are target genes of miR-142-5p and miR-130a-3p, respectively.

Because miRNAs may inhibit target gene expression by blocking protein translation or by degrading mRNA, we evaluated whether miR-142-5p or miR-130a-3p influences the mRNA and protein expression of their target genes. Transduction of the untreated macrophages with miR-142-5p mimics decreased SOCS1 mRNA and protein expression, whereas transduction of the M(IL-4) with miR-142-5p ASO increased SOCS1 mRNA and protein expression (Fig. 4c,d). Furthermore, inhibiting miR-130a-3p expression in resting macrophages with ASO enhanced PPARγ mRNA and protein levels, while miR-130a-3p mimics reduced PPARγ mRNA and protein levels in M(IL-4) (Fig. 4e,f). Thus, miR-142-5p and miR-130a-3p reduce the mRNA stability of SOCS1 and PPARγ mRNA in macrophages, respectively.

Because miRNAs may participate in signal transduction feedback circuits by maintaining the expression of signalling proteins^23^, we further evaluated the contributions of miR-142-5p and miR-130a-3p in maintaining SOCS1 and PPARγ levels in M2 macrophages by examining the kinetics of the miRNAs and their target genes in macrophages following IL-4 treatment. Consistent with previous findings that IL-4-induced SOCS1 provides negatively feedback to inhibit the STAT6 pathway^2^, IL-4 induced STAT6 phosphorylation at 1 h following treatment; this phosphorylation was markedly diminished at 8 h when the SOCS1 protein levels reached its peak. Interestingly, miR-142-5p gradually increased following IL-4 stimulation, peaked at 24 h, and remained at a high level for at least 48 h (Fig. 4g). In parallel, SOCS1 protein levels gradually decreased as miR-142-5p expression levels increased, recovering STAT6 phosphorylation at 24 h after IL-4 stimulation that was sustained for at least 48 h (Fig. 4g). In contrast, IL-4 reduced miR-130a-3p expression at 8 h, this expression fell to the lowest point by 24 h and remained at a low level for 48 h (Fig. 4h). Aligned with the kinetics of miR-130a-3p reduction, PPARγ expression increased by 8 h and was maintained at a high level for 48 h (Fig. 4h). Therefore, miR-142-5p upregulation and miR-130a-3p downregulation in M2 macrophages help to maintain M2 signalling by altering SOCS1 and PPARγ expression.

**Figure 5:**
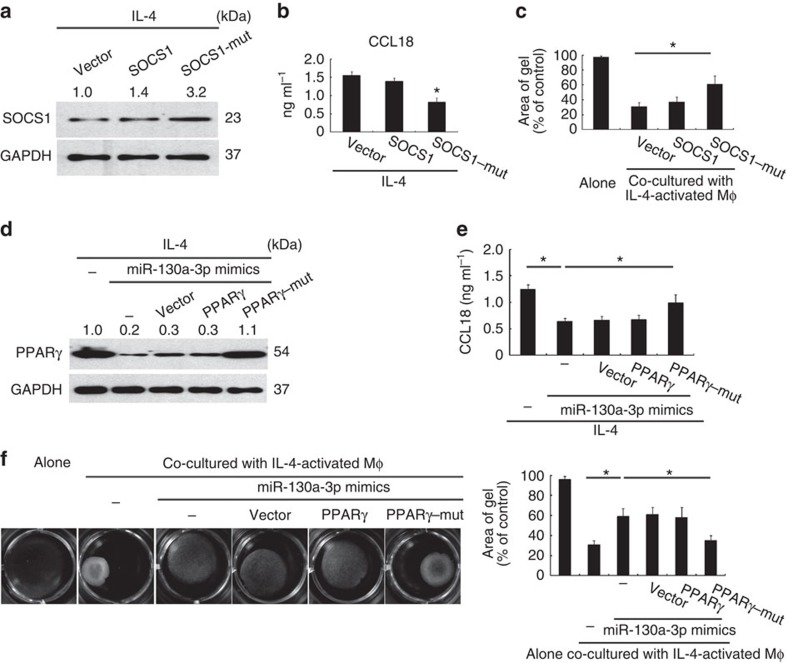
miR-142-5p and miR-130a-3p regulate profibrogenesis by targeting SOCS1 and PPARγ respectively. (**a**–**c**) MM6 cells were transfected with the pcDNA 3.1 plasmid vector or vectors cloned with a wild-type SOCS1 or mutated SOCS1 expression cassette at miR-142-5p (SOCS1-mut) response element. After 24 h, the cells were stimulated with IL-4 for 12 h and co-cultured with primary human fibroblasts for another 48 h. (**a**) Representative western blot of SOCS1 in MM6 cells (*n*=3). The numbers above the blot present the intensity ratio of SOCS1/GAPDH analysed by ImageJ. (**b**) CCL18 level in the media of MM6 cells (mean±s.e.m., *n*=3; *P<0.05 compared with cells transfected with vector by two-tailed Student's *t*-test). (**c**) Human primary fibroblast contractility in three-dimensional collagen matrices (mean±s.e.m., *n*=3 independent experiments; *P<0.05 by a two-tailed Student's *t*-test). (**d**–**g**) MM6 cells were transfected with miR-130a-3p mimics or co-transfected with a wild-type PPARγ or mutated PPARγ expression cassette at miR-130a-3p response element. After 24 h, the macrophages were stimulated with IL-4 for 12 h and co-cultured with primary human fibroblasts for another 48 h. (**d**) Representative western blotting for PPARγ in MM6 cells (*n*=3) (**e**) CCL18 level in the media of MM6 cells (mean±s.e.m., *n*=3; *P<0.05 by two-tailed Student's *t*-test). (**f**) Human primary fibroblast contractility in three-dimensional collagen matrices (mean±s.e.m., *n*=3 independent experiments; *P<0.05 by two-tailed Student's *t*-test).

To validate whether IL-4-induced miR-142-5p upregulation results in M2 polarization and profibrogenesis by silencing SOCS1, we knocked down SOCS1 in M(IL-4) that were transduced with miR-142-5p ASO (Supplementary Fig. 4a). Silencing SOCS1 recapitulated the capacity of M(IL-4) to produce M2 cytokines (Supplementary Fig. 4b) and to promote fibrogenesis (Supplementary Fig. 4c,d), which was suppressed by miR-142-5p ASO. To further confirm that miR-142-5p targets SOCS1 to maintain M2 macrophage activation, we rescued SOCS1 expression by transfecting M(IL-4) with a SOCS1 expression vector carrying a mutated seed sequence for miR-142-5p (SOCS1-mut) at its 3′ UTR. Transfection with SOCS1-mut, but not wild-type SOCS1 increased SOCS1 protein expression and decreased M2 cytokine production and profibrogenic ability in M(IL-4) (Fig. 5a–c).

We further determined whether elevated PPARγ expression due to reduced miR-130-3p expression contributes to M2 polarization in M(IL4). PPARγ elevation in M(IL4) was inhibited by rescuing miR-130-3p expression with miRNA mimics, but was retrieved by co-transfection with PPARγ-mut carrying a mutated miR-130-3p seed sequence at its 3′ UTR, but not with a wild-type PPARγ vector (Fig. 5d). Moreover, recovering PPARγ expression in M(IL-4) that were transfected with miR-130-3p mimics retrieved M2 polarization (Fig. 5e) and the profibrogenic effects of the macrophages (Fig. 5f). Collectively, increased miR-142-5p and reduced miR–130a-3p expression result in M2 polarization and profibrogenesis by silencing SOCS1 and by relieving PPARγ targeting, respectively.

### miR-142-5p and 130a-3p mediate Mϕ profibrogenesis in mice

**Figure 6:**
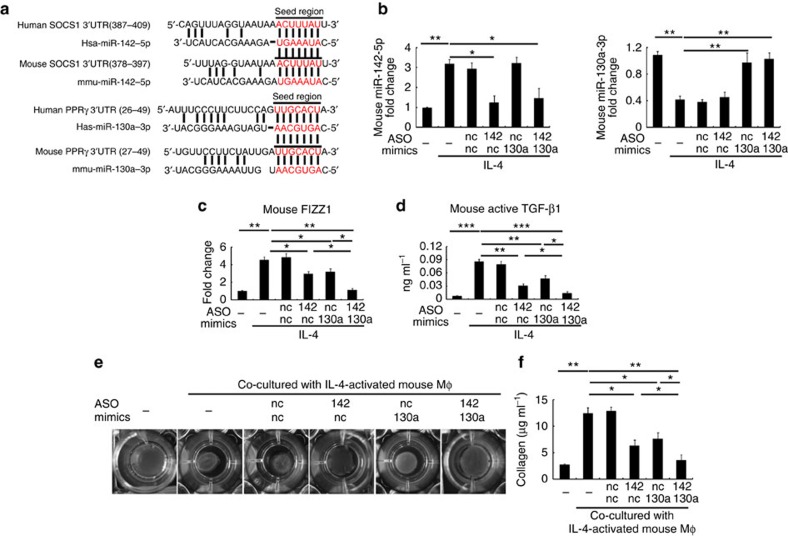
miR-142-5p and miR-130a-3p regulate profibrogenesis of mouse macrophages. (**a**) Humans and mice share conserved miR-142-5p-binding sites in the 3′UTR of SOCS1 mRNA and conserved miR-130a-3p-binding sites in the 3′UTR of PPARγ. (**b**) Primary mouse macrophages were transduced with nc, miR-142-5p ASO or miR-130a-3p mimics or both. After 24 h, the cells were stimulated with IL-4 for 24 h and expression of miR-142-5p and miR-130a-3p was examined by qRT–PCR (mean±s.e.m., *n*=4 independent experiments; *P<0.05; **P<0.01. *P* values were obtained using two-tailed Student's *t*-tests). (**c**,**d**) FIZZ1 mRNA expression (**c**) and active TGF-β1 production (**d**) in mouse macrophages treated as in (**b**) were examined by qRT–PCR and ELISA, respectively (mean±s.e.m., *n*=4 independent experiments; *P<0.05; **P<0.01; ***P<0.001. *P* values were obtained using two-tailed Student's *t*-tests). (**e**,**f**) Mouse macrophages were transfected with control, miR-142-5p ASO or miR-130a-3p mimics or both. After 24 h, the macrophages were stimulated with IL-4 for 12 h and co-cultured with mouse primary fibroblasts for another 48 h. The contractility in three-dimensional collagen matrices (**e**) and extracellular acid-soluble collagen production (**f**) of fibroblasts were examined (mean±s.e.m., *n*=4 independent experiments; *P<0.05; **P<0.01; ***P<0.001. *P* values were obtained using two-tailed Student's *t*-tests). nc, negative control.

Because SOCS1 and PPARγ are also key regulators of M2 polarization in mice^2^, we evaluated the contributions of miR-142-5p and miR-130a-3p in mouse M2 activation. The seed sequence of miR-142-5p at SOCS1-3′ UTR and that of miR-130a-3p at PPARγ-3′ UTR are conserved among humans and mice (Fig. 6a). Consistent with our findings in human macrophages, IL-4 increased miR-142-5p expression and reduced miR-130a-3p expression in mouse macrophages (Fig. 6b). Transduction of miR-142-5p ASO and miR-130a-3p mimics in IL-4-treated mouse macrophages synergistically inhibited M2 polarization (Fig. 6c,d) and their profibrogenic effect (Fig. 6e,f). Collectively, our data suggest that miR-142-5p and miR-130a-3p mediate M2 polarization and profibrogenic effects in both humans and mice.

### IL-4 upregulates miR-142-5p via STAT6

**Figure 7:**
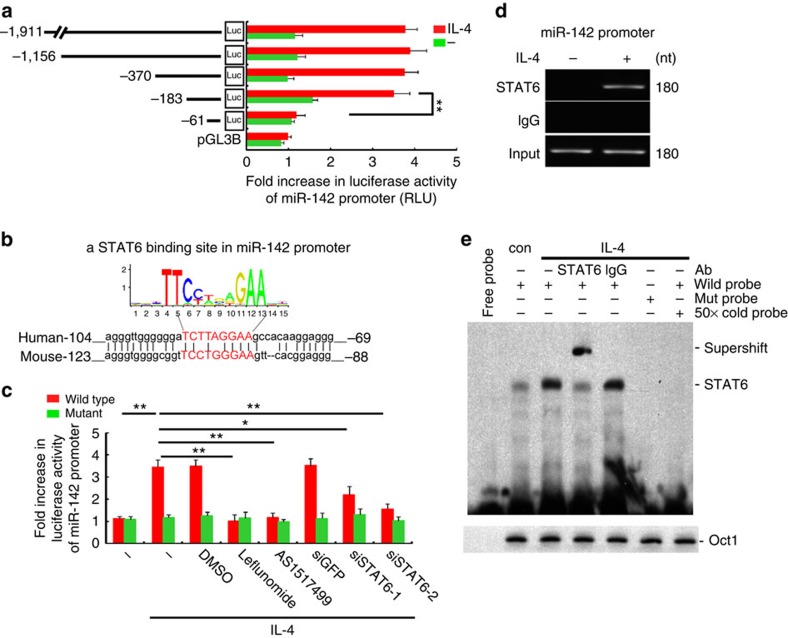
IL-4 upregulates miR-142-5p via STAT6. (**a**) MM6 cells were transfected with whole length or several deletion miR142-luc constructs, incubated with IL-4 and harvested for the luciferase activity assay(mean±s.e.m., *n*=3; **P<0.01 by two-tailed Student's *t*-test). (**b**) A conserved STAT6-binding element on the miR-142 promoter across species predicted by JASPAR. (**c**) MM6 cells were transfected with a wild-type or mutant reporter construct of miR-142, exposed to IL-4 with or without pretreatment with DMSO or STAT6 inhibitors or pretransfection with GFP-siRNA or STAT6-siRNAs, and harvested for the luciferase assay (mean±s.e.m, *n*=3; *P<0.05, **P<0.01 by two-tailed Student's *t*-test). (**d**) Lysate of human macrophages with indicated treatments was prepared for the ChIP assay using anti-STAT6 Ab or control IgG (*n*=3). (**e**) The nuclear extract of human macrophages with indicated treatments was prepared for EMSA. A mutated probe for STAT6, a competition experiment using 50-fold unlabelled STAT6 oligonucleotide and a supershift experiment with anti-STAT6 Ab were performed. Oct-1 is used as a loading control (*n*=2). RLU, relative light units.

Next, we investigated the mechanisms by which IL-4 upregulates miR-142-5p but downregulates miR-130a-3p. A series of pGL3 reporter plasmids carrying sequential deletions of the 5'-flanking region of miR-142-5p cloned upstream of the firefly luciferase gene were tested for IL-4 responsiveness upon transfected into Mono Mac6 (MM6) cells, a human macrophage cell line previously used for M2 gene transcription studies^6^ Sequence deletions from −1911 up to −183 bp upstream of the transcription start site (TSS) of miR-142 did not influence IL-4-induced luciferase activities, while deletion to −61 bp completely abolished these IL-4-induced effects (Fig. 7a). Sequence analysis revealed a STAT6-binding site conserved among humans and mice located at −91 to −83 bp upstream of the miR-142 TSS (Fig. 7b), wherein mutation of three nucleotides, treatment with STAT6 inhibitors (leflunomide and AS1517499) or transfection with STAT6-siRNA abrogated the IL-4-induced luciferase activities (Fig. 7c).. Binding of STAT6 to this motif was further confirmed by chromatin immunoprecipitation (ChIP) analysis using an anti-STAT6 antibody and IL-4-treated macrophages (Fig. 7d). In addition, electrophoretic mobility shift assay (EMSA) using 3'-biotin-labelled DNA probe containing the STAT6-binding site of the miR-142-5p promoter revealed an apparent DNA-protein complex when the nuclear extracts of IL-4-treated macrophages were applied (Fig. 7e). These data suggested that IL-4/STAT6 directly regulate miR-142 transcription via its promoter.

### IL-4 downregulates miR-130a-3p by histone deacetylation

**Figure 8:**
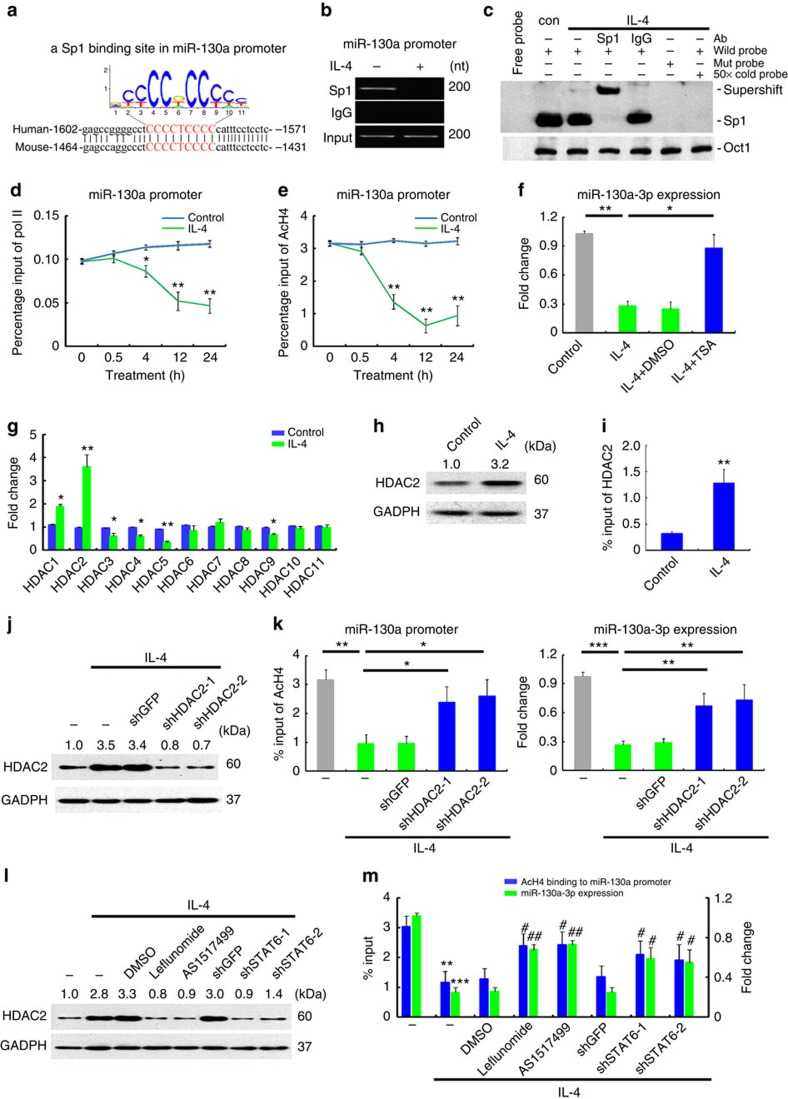
IL-4 downregulates miR-130a-3p by inducing histone deacetylation. (**a**) A conserved Sp1-binding element on the miR-130a promoter across species predicted by JASPAR. (**b**) Lysates of human macrophages with indicated treatments were prepared for the ChIP assay using anti-Sp1 Ab (*n*=3). (**c**) The nuclear extract of human macrophages with indicated treatments was prepared for EMSA(*n*=3). (**d**,**e**) Human macrophages were stimulated with IL-4 for indicated time and kinetics of RNA pol II (**d**) or AcH4 (**e**) on the miR-130a promoter were analysed by ChIP. The results are presented as enrichment (percentage of input DNA) of RNA pol II or AcH4 promoter occupancy(*n*=3). (**f**) Human macrophages were stimulated with IL-4 with or without pretreatment of DMSO or TSA.miR-130a expression was determined by qRT–PCR 24 h afterwards (*n*=3). (**g**) Human macrophages were stimulated with IL-4 for 24 h. The expression of the indicated HDACs was quantitated by qRT–PCR(*n*=3). (**h**) Human macrophages were stimulated with IL-4 for 48 h and the protein level of HADC2 was determined by western blot(*n*=3). (**i**) Binding of HDAC2 on the miR-130a promoter in human macrophages was analysed by ChIP(*n*=3). (**j**) Representative western blotting for HADC2 of IL-4-treated human macrophages transduced with HADC2-shRNAs(*n*=3). (**k**) Binding of AcH4 on the miR-130a promoter was analysed by ChIP (left) and the expression of miR-130a-3p (right) was analysed by qRT–PCR in cells treated as in (**j**) (*n*=3). (**l**) Representative western blotting for HADC2 of IL-4-treated human macrophages pretreated with STAT6 inhibitors or transduced with STAT6-shRNAs (*n*=3). (**m**) Binding of AcH4 on the miR-130a promoter was analysed by ChIP and expression of miR-130a-3p was analysed by qRT–PCR in cells treated as in (**l**) (*n*=3). (mean±s.e.m.; *P<0.05; **P<0.01 compared with control. ^#^*P*<0.05, ^#^*P*<0.01 compared with M(IL-4) for all the experiments above. The numbers above the blots present the intensity ratio of the indicated protein/GAPDH analysed by ImageJ).

Similarly, using a series of pGL3 reporter plasmids containing various deleted (Supplementary Fig. 5a) or mutated (Supplementary Fig. 5b) 5'-flanking regions of miR-130a, we identified a Sp1-binding site for miR-130a (Fig. 8a). Moreover, inhibitors of Sp1 (Supplementary Fig. 5b) efficiently abrogated the luciferase activities of the miR-130a promoter. Because the activator of M1 macrophages, LPS, enhances the nuclear translocation of Sp1, leading to overexpression of its target genes^24^, we speculated that IL-4 might inhibit Sp1 signalling and subsequently repress miR-130a expression in M2 macrophages. Interestingly, although IL-4 reduced Sp1 binding to the miR-130a promoter (Fig. 8b), nuclear translocation of the transcription factor remained unchanged upon IL-4 treatment (Fig. 8c and Supplementary Fig. 5c). Furthermore, ChIP analysis using RNA pol II antibody demonstrated that IL-4-reduced chromatin accessibility at the miR-130a promoter (Fig. 8d).

We further evaluated whether IL-4-mediated reduction in chromatin accessibility at the miR-130a promoter was a result of DNA methylation or histone modification. We identified few CpG islands at the promoter of miR-130a (Supplementary Fig. 5d), and treatment with 5-Aza-dC, a DNA methyltransferase inhibitor, failed to restore miR-130a expression in IL-4-treated macrophages (Supplementary Fig. 5e), excluding the contribution of DNA methylation to reduced miR-130a in M2 macrophages. Next, we examined histone modifications on the miR-130a-3p promoter by ChIP using AcH4 and H3K4me3 as markers of open chromatin and H3K9me3 and H3K27me3 as markers of closed chromatin. We observed that IL-4 decreased histone H4 acetylation (Fig. 8e), but did not change histone methylation (Supplementary Fig. 5f). TSA, an inhibitor of class I and II HDACs, relieved the IL-4-mediated inhibition of miR-130a transcription (Fig. 8f), suggesting that histone deacetylation is responsible for reduced accessibility of Sp1 to the promoter region of miR-130a.

Next, we further evaluated whether aberrant HDAC expression is responsible for histone deacetylation of miR-130a promoter in M(IL-4). IL-4 increased the mRNA expression of both HDAC-1 and 2, but not the other HDAC family members in the macrophages (Fig. 8g) as determined by qRT–PCR. However, IL-4 only enhanced the protein level of HDAC-2 (Fig. 8h), not HDAC-1 (Supplementary Fig. 5g). To further investigate whether HDAC-2 mediates IL-4-induced histone deacetylation of the miR-130a promoter, we examined the binding of HDAC2 to the miR-130 promoter by ChIP assay and found that IL-4 increased HDAC2 enrichment on the miR-130 promoter (Fig. 8i). Furthermore, silencing HDAC2 in M(IL-4) increased histone acetylation at the miR-130 promoter and miR-130a-3p expression (Fig. 8j,k), suggesting that IL-4 induces histone deacetylation of the miR-130a promoter by enhancing HDAC2 expression.

Because IL-4 stimulates STAT6 signalling in macrophages, we further investigated whether STAT6 signalling is responsible for HDAC2 upregulation. Treatment with STAT6 inhibitors or transduction with STAT6-shRNA in M(IL-4) dramatically reduced HDAC2 expression (Fig. 8l), reversed histone deacetylation at the miR-130a promoter and rescued miR-130a-3p expression (Fig. 8m). Collectively, miR-130a-3p transcription in resting macrophages is maintained by Sp1 but is inhibited in M(IL-4) by histone deacetylation at its promoter, which is mediated by STAT6-induced HDAC2.

### Dyregulated Mϕ miR-142-5p and 130a-3p enhance liver fibrosis

**Figure 9:**
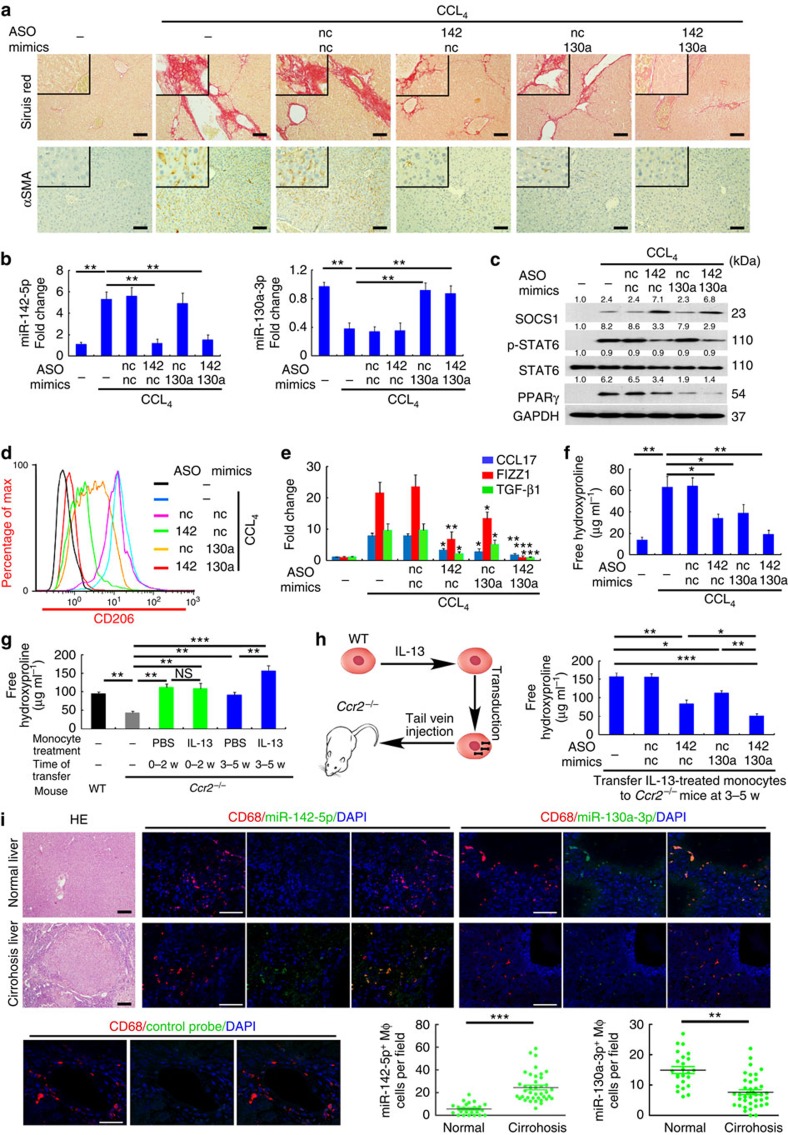
Dyregulated Mϕ miR-142-5p and miR-130a-3p enhance liver fibrosis. (**a**–**f**) Mice were intravenously injected with LNA-modified miR-142-5p ASO, miR-130a-3p mimic or both every 3 days after CCL_4_ challenge. Mice were sacrificed after 6 weeks (mean±s.e.m., *n*=8 mice/group; *P<0.05, **P<0.01 by two-tailed Student's *t*-test). (**a**) Representative images of Sirius Red staining and α-SMA immunochemical staining of mouse liver sections. Inserts show higher magnification. Scale bar, 50 μm. (**b**) Expression of miR-142-5p and miR-130a-3p in hepatic macrophages was evaluated by qRT–PCR. (**c**) SOCS1 level, STAT6 phosphorylation and PPARγ level in hepatic macrophages were determined by western blot analysis (*n*=3). (**d**) Expression of CD206 in hepatic macrophages was evaluated by flow cytometry analysis. (**e**) Expression of CCL17, FIZZ1 and TGF-β1 in hepatic macrophages was evaluated by qRT–PCR. (**f**) Hydroxyproline in the livers of mice was measured by the Sircol assay (**g**) C57BL/6 wild-type or *Ccr2*^−/−^ mice were gavaged with CCL_4_ every 5 days for 6 weeks.1 × 10^6^ bone marrow-derived monocytes were treated with IL-13 for 24 h, and injected intravenously into CCL_4_-treated *Ccr2*^−/−^ mice either at weeks 0, 1, and 2 (0–2 weeks) or at weeks 3, 4 and 5 (3–5 weeks) of treatment. Hydroxyproline was measured in the livers 6 weeks after CCL_4_ challenge (mean±s.e.m., *n*=8 mice/group *P*<0.05, **P<0.01, ***P<0.001 by two-tailed Student's *t*-test). (**h**) Bone marrow-derived monocytes were transduced with control, miR-142-5p ASO or miR-130a-3p mimics or both. After 24 h, the monocytes were stimulated with IL-13 for 24 h and injected intravenously into CCL_4_-treated *Ccr2*^−/−^ mice at weeks 3–5 of treatment. (left) Schematics of experimental designs. (right) Mice were killed 6 weeks after CCL_4_ challenge and hydroxyproline was measured in the livers. (Mean±s.e.m., *n*=8 mice/group *P*<0.05, **P<0.01, ***P<0.001 by two-tailed Student's *t*-test.) (**i**) Representative H&E staining, FISH for miR-142-5p, miR-130a-3p or scramble control and co-immunostaining for CD68 in normal and cirrhotic human liver tissues. Scale bars, 50 μm. Quantification of miR-142-5p^+^ and miR-130a-3p^+^macrophages (normal liver group, *n*=24; cirrhotic liver group, *n*=39, mean±s.e.m., **P<0.01, ***P<0.001 by two-tailed Student's *t*-test.).

Hepatic macrophages underlie the progression of liver cirrhosis by activating hepatic fibroblasts in mice^25^ and in humans^26^. Therefore, we investigated whether modulating miR-142-5p and miR-130a-3p would influence the progression of mouse liver fibrosis. We used locked nucleic acid (LNA)-modified miR-142-5p ASO or/and miR-130a-3p mimic that are more stable *in vivo* and with less off-target effects^27^,^28^. Corn oil gavage with carbon tetrachloride (CCL_4_) in mice resulted in massive liver fibrosis (Fig. 9a,f) as reported previously^25^, and led to increased miR-142-5p expression and decreased miR-130a-3p expression in the isolated hepatic macrophages (Fig. 9b). Additionally, STAT6 phosphorylation and PPARγ expression were enhanced in the hepatic macrophages isolated from the mice (Fig. 9c), accompanied by increased expression of profibrotic markers including CD206 (Fig. 9d), CCL17, FIZZ1 and TGF-β1 (ref. 1, 8, 29, 30) (Fig. 9e). However, intravenous injection of LNA-modified miR-142-5p ASO or miR-130a-3p mimics following CCL_4_ challenge reduced miR-142-5p and increased miR-130a-3p in the hepatic macrophages to normal ranges (Fig. 9b), consistent with previous findings that LNA-modified oligonucleotides alter miRNA expression in immune cells *in vivo*^27^,^28^. More importantly, treatment with miR-142-5p ASO elevated SOCS1 levels and reduced STAT6 phosphorylation, while treatment with miR-130a-3p mimics repressed PPARγ expression (Fig. 9c). Additionally, either of these treatments substantially inhibited profibrotic marker expression in hepatic macrophages, alleviated liver fibrosis as determined by Sirius Red staining, suppressed hepatic fibroblast activation as evaluated by α-SMA staining, and reduced the level of the collagen-specific amino acid hydroxyproline (Fig. 9a,d–f). Furthermore, combined treatment with both miR-142-5p ASO and miR-130a-3p mimics almost completely abrogated profibrotic marker expression in hepatic macrophages and liver fibrosis (Fig. 9a–f).

Next, we further investigated whether alterations of miR-142-5p and miR-130a-3p in macrophages contribute to hepatic fibrogenesis using chemokine receptor-2–deficient (*Ccr2*^−/−^) mice, which have been widely used to study the particular roles of monocyte/macrophages in lung and liver fibrogenesis^31^,^32^,^33^. Upon challenge with CCL_4_, *Ccr2*^*−/−*^ mice showed markedly reduced liver fibrosis compared with the wild-type mice. However, transferring either monocytes or M(IL-13) into the *Ccr2*^−/−^ mice recapitulated hepatic fibrosis, with M(IL-13) transfer at a later stage (between 3 to 5 weeks following the CCL_4_ challenge) showing the most prominent profibrogenic effects (Fig. 9g). More importantly, transferring M(IL-13) that were transduced with either miR-130a-3p mimics or miR-142-5p ASO into the CCL4-treated *Ccr2*^−/−^ mice rendered much milder liver fibrosis compared with transferring the untransduced M(IL-13) cells. Furthermore, transducing the monocytes with both miRNAs completely abrogated their profibrogenic effects upon transfer into CCL_4_-treated *Ccr2*^−/−^ mice (Fig. 9h). Importantly, administration of LNA-modified miR-142-5p ASO and miR-130a-3p mimic failed to inhibit CCL_4_-induced liver fibrosis in *Ccr2*^*−/−*^ mice (Supplementary Fig. 6a). Collectively, modulating the expression of miR-142-5p and miR-130a-3p in hepatic macrophages inhibits the progression of liver fibrosis.

To determine the clinical relevance of our findings, we examined the expression of miR-142-5p and miR-130a-3p using fluorescence *in situ* hybridization (FISH) in the liver samples of patients with hepatitis B induced liver cirrhosis. Co-immunostaining of CD68 was used to denote macrophages in the same sections. miR-142-5p expression was significantly increased in the macrophages of the cirrhotic liver tissues compared with normal liver tissues resected from patients with hepatic haemangioma, while miR-130a-3p expression was markedly decreased (Fig. 9i), demonstrating the correlation of altered miR-142-5p and miR-130a-3p expression in hepatic macrophages of liver cirrhosis.

### Dyregulated Mϕ miR-142-5p and 130a-3p enhance lung fibrosis

**Figure 10:**
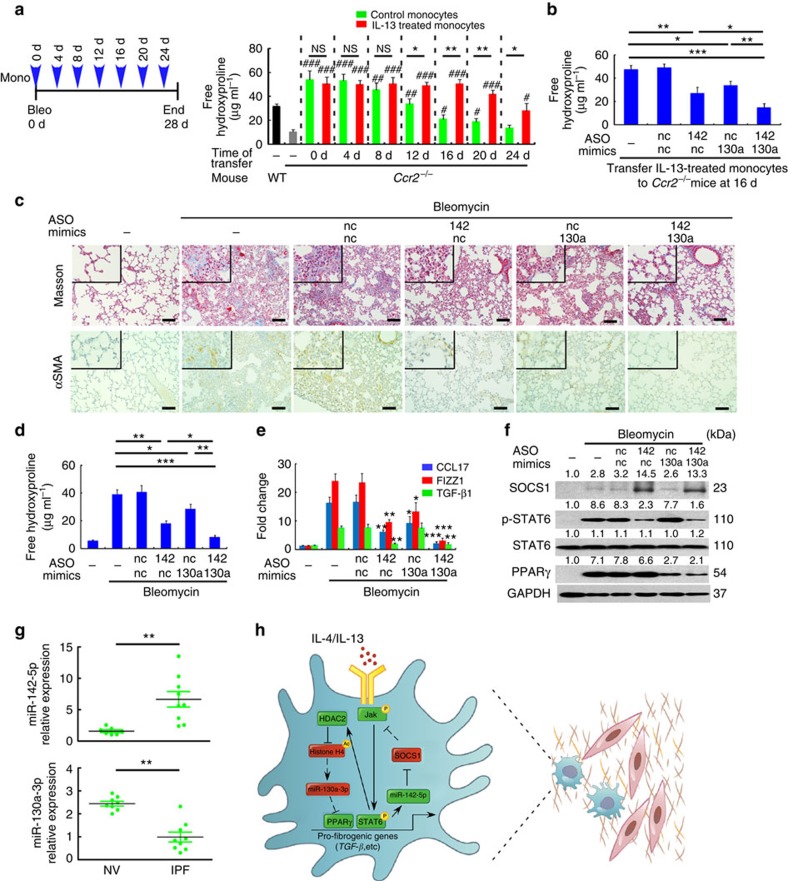
Dyregulated Mϕ miR-142-5p and miR-130a-3p enhance lung fibrosis. (**a**) C57BL/6 wild-type or *Ccr2*^−/−^ mice were intratracheally administered 0.15 U bleomycin.1 × 10^6^ bone marrow-derived monocytes were treated with IL-13 for 24h, and injected intravenously into bleomycin-treated *Ccr2*^−/−^ mice either at days 0, 4, 8,12,16,20 or 24 after the treatment. (left) Schematics of experimental designs. (right) Hydroxyproline was measured in the lungs 28 days after bleomycin challenge. (mean±s.e.m., *n*=8 mice/group; ^#^*P*<0.05, ^##^*P*<0.01, ^###^*P*<0.001 compared with *Ccr2*^−/−^ mice without monocyte transfer.*P<0.05, **P<0.01 by two-tailed Student's *t*-test). (**b**) Bone marrow-derived monocytes were transduced with control, miR-142-5p ASO or miR-130a-3p mimics or both. After 24 h, the monocytes were stimulated with IL-13 for 24 h and injected intravenously into *Ccr2*^−/−^ mice 16 days after bleomycin challenge. Mice were killed 28 days after bleomycin challenge, and hydroxyproline was measured in the lungs (mean±s.e.m., *n*=8 mice/group; *P<0.05, **P<0.01, ***P<0.001 by two-tailed Student's *t*-test). (**c**–**f**) C57BL/6 wild type were intravenously injected with LNA-modified miR-142-5p ASO, miR-130a-3p mimic or both 16 days following intratracheal administration of bleomycin and repeated every 3 days. Mice were killed 28 days after the bleomycin challenge (mean±s.e.m., *n*=8 mice/group; *P<0.05, **P<0.01 by two-tailed Student's *t*-test). (**c**) Representative images of Masson trichrome staining and α-SMA immunostaining of mouse lung sections. Inserts show higher magnification. Scale bar, 50 μm. (**d**) Hydroxyproline in the lungs of mice was measured by the Sircol assay. (**e**) Expression of CCL17, FIZZ1 and TGF-β1 in pulmonary macrophages was evaluated by qRT–PCR. (**f**) SOCS1 level, STAT6 phosphorylation and PPARγ level in pulmonary macrophages were determined by western blot analysis (*n*=3). (**g**) Expression of miR-142-5p and miR-130a-3p in macrophages from BAL fluids of normal volunteers and IPF patients was evaluated by qRT–PCR (normal volunteers group, *n*=7; IPF patients group, *n*=9; mean±s.e.m., **P<0.01 by two-tailed Student's *t*-test). (**h**) Schematics highlighting the primary findings of this study. IL-4/IL-13 increases miR-142-5p via STAT6 and reduces miR-130a-3p by histone deacetylation in macrophages. These miRNAs target SOCS1 and PPARγ, respectively, and modulate a profibrogenic macrophage program.

The *in vivo* functions of macrophage-related miR-142-5p and miR-130a-3p were also studied in wild-type and *Ccr2*^−/−^ mouse models of lung fibrosis induced by bleomycin^31^,^32^. The role of macrophages in lung fibrosis was confirmed as *Ccr2*^−/−^ mice showed markedly reduced lung fibrosis compared with wild-type mice upon challenged by bleomycin, while transferring of monocytes or M(IL-13) recapitulated lung fibrosis in the *Ccr2*^−/−^ mice. Moreover, transferring M(IL-13), particularly from 12 to 24 days following bleomycin challenge, generated much stronger lung fibrosis compared with transferring unactivated monocytes (Fig. 10a). These data suggest that Th2 signalling confers profibrogenic effects on macrophages, which lead to lung fibrosis at a later stage after injury. To study the particular roles of miR-142-5p and miR-130a-3p in profibrotic macrophages in *vivo*, we transduced miR-142-5p ASO or/and miR-130a-3p mimics to wild-type M(IL-13) and transferred these cells to *Ccr2*^−/−^ mice challenged with bleomycin. Similar to their effects on hepatic fibrogenesis, the transduction of miR-130a-3p mimics and miR-142-5p ASO to M(IL-13) synergistically abrogated the profibrotic effects of these cells in the lungs (Fig. 10b).

To further explore the therapeutic potentials of LNA-modified oligonucleotides in lung fibrosis, we administered bleomycin intratracheally into wild-type mice and initiated intravenous injection of LNA-modified miR-142-5p ASO or/and miR-130a-3p mimic 16 days after bleomycin challenge, which was then repeated every 3 days until 28d, when the animals were sacrificed for examination. Similar to the findings in liver fibrosis, administering miR-142-5p ASO and miR-130a-3p mimics significantly reduced the degree of lung fibrosis in the bleomycin-treated mice (Fig. 10c,d). This finding was consistent with marked decreases in profibrotic marker expression (Fig. 10e), STAT6 phosphorylation and PPARγ expression (Fig. 10f) in the pulmonary macrophages of the treated mice. In contrast, LNA-modified miR-142-5p ASO and miR-130a-3p mimic did not exert anti-fibrotic effects against bleomycin-induced lung lesion in *Ccr2*^*−/−*^ mice (Supplementary Fig. 6b). Therefore, our findings suggested the therapeutic potential of inhibiting miR-142-5p and elevating miR-130a-3p in macrophages to treat lung fibrosis.

To determine whether miR-142-5p and miR-130a-3p are involved in human IPF, we isolated macrophages from the bronchial alveolar lavage (BAL) fluid of normal volunteers and IPF patients and examined the expression of miR-142-5p and miR-130a-3p by qRT–PCR. Consistent with our findings in mouse lung fibrosis, miR-142-5p expression in the macrophages of BAL fluid from IPF patients was increased, while miR-130a-3p expression was reduced (Fig. 10g). Taken together, these results indicated that aberrant miR-142-5p and miR-130a-3p expression in lung macrophages might be involved in the development of pulmonary fibrosis.

## Discussion

Although miscellaneous pathological processes may lead to fibrosis, the Th2 response induced by pathogen infections, such as hepatitis viruses, and autoimmune attack, such as IPF, is a crucial link between inflammation and fibrosis^5^,^34^. In the milieu dominant in the Th2 response, macrophages activated by Th2 cytokines drive fibrogenesis by producing potent profibrogenic cytokines^5^,^6^,^9^,^18^,^35^. However, negative feedback circuits are formed during exposure to Th2 cytokines to deactivate the profibrogenic signalling pathway^2^. Because fibrosis is a chronic pathological process that requires continuing profibrogenic signals, macrophages must maintain profibrogenic polarization to perpetuate fibrogenesis. Here, we demonstrated that increased miR-142-5p and reduced miR-130a-3p in macrophages upon Th2 stimulation help to maintain M2 polarization and their profibrogenic activities. Mechanistically, the elevated miR-142-5p prolongs STAT6 phosphorylation by targeting SOCS1, while miR-130a reduction relieves its repression of PPARγ, a STAT6 coordinator. Furthermore, changes in the levels of both miRNAs are critical to the pathogenesis of lung and liver fibrosis in mice and humans (Fig. 10h).

STAT6 is the principal transcriptional factor in mediating M2 gene expression^2^. Our data revealed that Th2 cytokine-induced miR-142-5p upregulation helps to maintain long-term STAT6 phosphorylation by silencing its negative regulator, SOCS1. SOCS1 is a target gene transcribed by STAT6 but that provides negatively feedback to inhibit STAT6 phosphorylation^2^. Our data showed that miR-142-5p upregulated by STAT6 suppresses SOCS1, a STAT6 inhibitor, as it reaches its peak of expression at later time points following Th2 stimulation, which is important for the persistent activation of the STAT6 pathway to maintain an M2 signature. Although the functions and targets of miR-142-5p have been not reported previously, the preferential expression of the miR-142 family in hematopoietic tissues with the highest expression in myeloid lineages^36^, indicates its role in immune regulation. Our present findings further clarify the role of miR-142 in immune cells as a maintainer of sustained M2 activation.

In addition to persistent STAT6 phosphorylation, intensive M2 polarization requires other coordinators^37^. PPARγ, a primary coordinator of STAT6, acts in concert with STAT6 to fully induce several hallmark M2 genes^38^. PPARγ is downregulated in M1 macrophages but upregulated in M2 cells^39^. Our study showed that miR-130a-3p targets PPARγ, while Th2 stimulation suppresses miR-130a-3p expression by inducing histone deacetylation at its promoter region, thus relieving its repression of PPARγ. In contrast, the M1 activator LPS upregulates miR-27b to silence PPARγ expression in M1 macrophages^17^, thus inhibiting M2 gene expression. Hence, differentially expressed miRNAs in distinctly polarized macrophages may play a role in maintaining their activation status. Moreover, our findings that blocking miR-142-5p with ASO together with increasing miR-130a-3p with miRNA mimics almost completely abrogated M2 activation and their profibrogenic effects support the finding that changes in the levels of multiple miRNAs may act synergistically to sustain macrophages in a certain activation status.

Previous studies have reported the contribution of miRNAs in fibrogenesis, however, these studies focused primarily on miRNA functions in fibroblasts. Among the studied miRNAs, miR-21 was shown to be upregulated in fibroblasts by TGF-β and to promote fibrogenesis in bleomycin-induced pulmonary fibrosis models and IPF patients^40^. Nevertheless, in addition to TGF-β, many other cytokines in the Th2 milieu may also contribute to tissue fibrosis^41^, and macrophages are a major cell type that secretes these mediators, such as CCL18 (ref. 7). Therefore, adjusting the polarization of macrophages may be more effective for preventing tissue fibrosis than for modulating the effect of a single cytokine. Indeed, administration of miR-142-5p ASO or/and miR-130a-3p mimics successfully prevented fibrosis in two major fibrotic disease mouse models. These findings may have clinical applications, as miR-142-5p is upregulated and miR-130a-3p is downregulated in the macrophages in livers of patients with liver cirrhosis and in those in the BAL fluid of IPF patients.

Different stimulators of macrophages may cause distinct miRNA expression profiles in various subsets of macrophages. Glioblastoma-infiltrating macrophages, which are activated primarily by M-CSF, display downregulated miR-142-3p compared with GM-CSF-treated cells^42^. Consistent with our findings, miR-142-5p, was markedly upregulated in renal fibrosis induced by unilateral ureteral obstruction^43^. The varied levels of miR-142 expression in different subtypes of activated macrophages may be due to the activation of different signalling pathways and to the induction of transcription factors by different stimulating cytokines. Our data showed that IL-4/IL-13 increase primary miR-142 transcription via STAT6 signalling, which is not a major signalling pathway of M-CSF^44^. Therefore, distinct miRNA expression in various activation subtypes of macrophages may underlie the differential functions of macrophages in fibrosis and malignant tumours.

Macrophages are known to play a crucial role in tissue fibrogenesis as selective macrophage depletion dramatically inhibited CCL_4_-induced liver fibrosis^33^,^45^ and bleomycin-induced lung fibrosis^46^,^47^. Profibrogenic macrophages are a critical source of TGF-β, which is a key driver of fibrosis^1^,^6^,^35^,^46^ However, different studies have identified distinct cytokines that are responsible for inducing profibrotic, TGF-β-producing macrophages. Some reports indicated that IL-4 and IL-13 are crucial mediators of bleomycin-induced pulmonary fibrosis^48^,^49^ and CCL_4_-induced liver fibrosis^50^, because depletion or blockade of IL-4, IL-13 and STAT6 markedly reduced the production of profibrogenic factors and fibrogenesis in these models^6^,^30^,^48^,^49^,^51^. However, other studies demonstrated that bleomycin/CCL_4_-mediated fibrosis results from proinflammatory cytokines, such as IL-17 and TNF-α (refs 30, 52). In fact, evidence supporting these differential observations is not mutually exclusive. First, different subsets of macrophages may contribute to fibrogenesis at different stages of injury. M2 macrophages play minor roles at the early stage (acute phase) of fibrosis but are essential to fibrogenesis at the later stage (progressive phase)^31^,^48^. Consistently, we observed that the profibrotic effects of IL-13-activated monocytes transferred to *Ccr2*^*−/−*^ mice were most pronounced at a later stage following bleomycin/CCL_4_ insults. Additionally, bleomycin/CCL_4_ may activate multiple signalling pathways in macrophages to induce TGF-β production and to exert their profibrogenic effects. Indeed, fibrogenesis driven by Th2 cytokines or proinflammatory mediators depends largely on TGF-β (refs 30, 52). TGF-β production was greatly enhanced when macrophages were treated with both IL-13 and TNF-a (ref. 6). Therefore, macrophages infiltrating chronic inflammatory lesions *in vivo* may share features of M1 and M2 subtypes and fall somewhere within a continuous spectrum between the binary M1/M2 definition^53^. Collectively, our data and those of others groups suggest that macrophages activated by Th2 cytokines, along with other mediators, play an essential role in the late stage of fibrosis.

Both monocytes and M(IL-13) transferred into CCL_4_/bleomycin-challenged *Ccr2*^−/−^ mice restored fibrosis in these knockout mice, while M(IL-13) transferred at a later stage showed the most prominent profibrogenic effects. However, whether the transferred M(IL-13) maintained their phenotype in the recipient mice remain unclear. Since macrophages are highly plastic in their polarization^53^, they could adopt a M1 phenotype in the inflammatory microenvironment of the CCL_4_/bleomycin-challenged recipients. Nevertheless, our data and those of another group^43^ indicated that miR-142-5p and miR-130a-3p were dysregulated in multiple organ fibrosis of both humans and mice. More importantly, inhibiting miR-142-5p and increasing miR-130a-3p expression with LNA-modified oligonucleotides alleviated both CCL_4_-induced liver fibrosis and bleomycin-induced lung fibrosis, highlighting the therapeutic importance of these miRNAs. TGF-β1 clearly plays a major role in these fibrosis models^1^,^6^ Our data showed that manipulation of these two miRNAs reduced TGF-β1 production by M(IL-4) and M(IL-13) *in vitro* and *in vivo*. Profibrogenic macrophages are a critical source of TGF-β (refs 35, 46). Indeed, the therapeutic effects of LNA-modified miR-142-5p ASO and miR-130a-3p mimics against fibrosis were absent in *Ccr2*^*−/−*^ mice. Collectively, our data suggested that the LNA-modified oligonucleotide treatments work primarily by blocking TGF-β1 production and activation by macrophages.

In summary, our study demonstrates a previously unappreciated miRNA regulatory network in macrophages as an important mechanism to perpetuate fibrogenesis. Future studies are warranted to further explore the translational values of these miRNAs as diagnostic markers and therapeutic targets for fibrosis in various chronic inflammatory diseases.

## Methods

### Macrophage isolation and culture

Peripheral blood mononuclear cells from healthy donors were isolated by Ficoll density gradient centrifugation^54^ The cells were plated in plastic 24-well plates (Costar, Cambridge, MA) at 5 × 10^6^ cells per well in DMEM alone (500 μl) and allowed to adhere for 2 h. The adherent cells were washed to remove the residual lymphocytes. Over 90% of adherent cells were monocytes as confirmed by FACS examination of CD14 and the starting number was ∼1 × 10^6^ cells per well. Macrophages were obtained by culturing monocytes in DMEM medium supplemented with 10% heat-inactivated human AB serum for 6 days and the macrophage yield was ∼5 × 10^5^ cells per well. In some experiments, macrophages were incubated with 100 ng ml^−1^ LPS, 500 U ml^−1^ IFN-γ, 20 ng ml^−1^ IL-4, 20 ng ml^−1^ IL-13, 20 ng ml^−1^ IL-10 or 5 ng ml^−1^ TGF-β (PeproTech, Rocky Hill, NJ) for 48 h. Bone marrow-derived macrophages of mice were obtained as described previously^55^. Briefly, femurs and tibias were removed from 4–8-week-old BALB/C mice, and bone marrow was flushed with sterile PBS. Bone marrow cells were plated in plastic 24-well plates (Costar) and cultured in 10% fetal bovine serum (FBS)/DMEM medium supplemented with 100 ng ml^−1^ mouse M-CSF (PeproTech). After 8 d, adherent cells were washed in PBS and then stimulated with 20ng ml^−1^ mouse IL-4 (PeproTech) for 48 h. This protocol yields 2–6 × 10^7^ macrophages per mouse (two femurs and two tibias) as determined by FACS examination of CD11b. All samples were collected with the informed consent of donors, and all related procedures were performed with the approval of the Internal Review and Ethics Boards of Sun Yat-Sen Memorial Hospital. All animal work was conducted in accordance with a protocol approved by the Institutional Animal Care and Use Committee at the Medical College of Sun Yat-Sen University.

### Fibroblast isolation and culture

Primary human fibroblasts were isolated from normal human breast samples obtained from reduction mammoplasties. Briefly, we incubated specimens in 5% FBS/DMEM containing 2 mg ml^−1^ collagenase I and 2 mg ml^−1^ hyalurinidase (Sigma-Aldrich, China) for 4 h. Fibroblasts are culture in 10% FBS/DMEM medium and passaged every 6–8 days. We used fibroblasts from passages four to nine. We isolated primary dermal mouse fibroblasts from BALB/C mice. The adherent outgrowth cells from minced skin were cultured in 10% FBS/DMEM medium. All samples were collected with the informed consent of the donors, and all related procedures were performed with the approval of the Internal Review and Ethics Boards of Sun Yat-Sen Memorial Hospital. All animal work was conducted in accordance with a protocol approved by the Institutional Animal Care and Use Committee at the Medical College of Sun Yat-Sen University.

### Co-culture experiments

Fibroblasts (2 × 10^5^) were added into the lower chamber and macrophages (5 × 10^5^) were added to the upper chamber of a 12-well transwell apparatus with 0.4 μm pore size (Costar, Cambridge, MA).The fibroblasts were subjected to the further analysis at 48 h after co-culturing.

### Three-dimensional collagen gels

Cell suspensions in serum-free medium were mixed with 3 mg ml^−1^ neutralized rat tail collagen type 1 at a ratio of 2:1. Then, cell suspensions at a density of 2 × 10^5^ cells ml^−1^ were seeded onto 24-well tissue culture plates and the gels were allowed to polymerize at 37 °C for 1 h before adding 1 ml of medium and detaching the edge of the gels from the walls of the well. After 48 h, images of the gels were taken and gel area were measured using ImageJ software (US National Institutes of Health).

### BrdU incorporation assay

We measured the proliferation of fibroblasts by a BrdU incorporation kit (Calbiochem, La Jolla, Ca) according to the manufacturer's instructions.

### Sircol assay for collagen

We assessed acid-soluble collagen in cell culture supernatants by Sircol dye binding assay kit (BioColor Ltd, Northern Ireland, UK) according to the manufacturer's instructions.

### MicroRNA expression

Total RNA from human primary macrophages treated with or without 20 ng ml^−1^ IL-4 or 20 ng ml^−1^ IL-13 for 48 h was isolated with a mirVana miRNA Isolation kit (Ambion, Foster, CA). After having passed RNA quantity measurement using the NanoDrop 1000, the samples were labelled using the miRCURY Hy3/Hy5 Power labelling kit and hybridized on the miRCURY LNA Array (v.18.0; covering 1921 human miRNAs, Exiqon,Vedbaek, Denmark). Following the washing steps the slides were scanned using the Axon GenePix 4000B microarray scanner. Scanned images were then imported into GenePix Pro 6.0 software (Axon) for grid alignment and data extraction. Replicated miRNAs were averaged and miRNAs that intensities ⩾30 in all samples were chosen for calculating normalization factor. Expressed data were normalized using the Median normalization. After normalization, differentially expressed miRNAs were identified through Fold Change filtering. qRT–PCR was performed using the miScript Reverse-Transcription Kit and miScript SYBR Green PCR Kit (Qiagen, China) on a Lightcycler 480 (Roche Diagnostics, China). The expression level of mature miRNAs was calculated using U6 as an internal control. Each sample was tested in triplicate. qPCR primers are from the Qiagen miScript Primer Assay as following: hsa-miR-142-5p (MS00006671), mmu-miR-142-5p (MS00006062), hsa-miR-130a-3p (MS00003444), mmu-miR-130a-3p (MS00001547), hsa-miR-651-5p (MS00005320), hsa-miR-210-3p (MS00003801), hsa-miR-379-5p (MS00009653), hsa-miR-521 (MS00033873), hsa-miR-377-3p (MS00004095), hsa-miR-34c-3p (MS00009548), hsa-miR-875-3p (MS00010619), hsa-miR-188-3p (MS00008897), has-U6 small nuclear RNA (MS00033740) and mmu-U6 small nuclear RNA (QT00117278) respectively. We determine the absolute level of miRNAs as previously reported^56^. Briefly, we generated standard curves by using serial dilutions of cDNA prepared from synthetic miRNAs with known concentrations(Takara Bio Company). Equivalent molecules per cell were extrapolated from the standard curve and threshold cycle (Ct) values, based on the assumption that total RNA per cell is 20 pg (ref. 56). The Gene Expression Omnibus database accession number is GSE59348 for miRNA microarray.

### Cell tranduction

Lentivirus packaging was made by GenePharma Inc (Shanghai, China). A lentiviral vector plasmids pGLV2-U6+puro was used in our study to construct the stable clones. The sequence information for miRNA cloning is provided in Supplementary Table 3 Virus was produced according to the user's manual (GenePharma). Primary human macrophages were transduced with the lentiviral vectors as previously described with slight modification^57^. Briefly, 1*10^6^ cells were transduced with the lentiviral vectors (multiplicity of infection[MOI] of 10) overnight at 37 °C with 4 μg ml^−1^ protamine sulfate (Sigma). Primary mouse macrophages were transfected with miRNA mimic or ASO using Amaxa Nucleofector technology from Lonza Cologne AG (Cologne, Germany) as previously described^17^. Briefly, 1 × 10^6^ cells were transfected with 200 pmol mouse miR-142-5p ASO or the scramble negative control and 10 pmol mouse miR-130a-3p or scramble negative control (Qiagen).

### Northern blot

Total RNA was loaded onto 2% formaldehyde/MOPS/agrose gels alongside an appropriate marker. After electrophoresis, RNA was transferred onto a positively charged nylon membrane (GE healthcare, Shanghai, China) for more than 6 h and prehybridized for 30 min at 50 °C in DIG Easy Hyb buffer (Roche). Locked nucleic acid oligonucleotides labelled with digoxin (miRCURY LNA Array detection probes, Exiqon) were hybridized to the membranes overnight at 42 °C. Afterward, membranes were washed with low stringency buffer (2 × SSC containing 0.1%SDS) at room temperature and later high stringency buffer (0.1 × SSC containing 0.1% SDS) at 42 °C, membranes were blocked with blocking solution (Roche) and incubated in antibody solution (Anti-Digoxigenin-AP, 1:10,000, Roche) for 30 min at room temperature. After washing in Maleic acid buffer containing 0.3% Tween 20, membranes were incubated with CSPD chemiluminescent solution and exposed on a Lumi-film X-ray film.

### ELISA assay

Human CCL18, human CCL13, human TGF-β 1 and mouse TGF-β 1 ELISA kits were from R&D Systems (Minneapolis, MN). Human CCL17 ELISA kit was from RayBiotech (Norcross, GA).

### Flow cytometry

Cells were stained with fluorochrome-conjugated monoclonal antibodies against human CD206 (Cat. No. 12–2069, eBioscience, San Diego, CA; 1.25 μg ml^−1^), human CD36 (Cat. No. 11–0396, eBioscience; 2.5 μg ml^−1^) and mouse CD206 (Cat. No. MA5–16870, Thermo; 2.5 μg ml^−1^), and subsequently analysed by multicolour flow cytometry using CellQuest software version 7.5.3 (FACSVantage-SE, BD Immunocytometry Systems, San Diego, CA).

### Fluid phase pinocytosis

Macrophages with indicated treatments were cultured in DMEM containing 3 mg ml^−1^ HRP (Sigma) for 2 h at 37 °C. After the macrophages were incubated, non-cell-bound enzymes were removed by four washes in ice-cold PBS. The collected cells were lysed with lysis buffer (150 mM NaCl, 0.1% Triton X-100, and 5% glycerol). The enzyme assay was conducted in a 96-well plate using *O*-phenylenediamine (Sigma) as the chromogenic substrate.

### Immunofluorescence

Cells were incubated with primary antibodies against fibronectin (sc-9068, Santa Cruz; 1:100) or FAP (AF3715, R&D Systems; 10 μg ml^−1^), followed by incubation with Alexa Fluor 488-conjugated secondary antibodies (Molecular Probes, Eugene, OR; 1:200). The fluorescence intensity was quantitated using ImageJ software.

### microRNA target prediction

The miRNA databases and target prediction tools miRanda-mirSVR (http://www.microrna.org/microrna/home.do), PicTar (http://pictar.mdc-berlin.de/) and TargetScan (http://www.targetscan.org/index.html) were used to identify potential miRNA targets. MiRanda and TargetScan predicted SOCS1 as the target gene of miR-142-5p, while all three miRNA target prediction databases predicted PPARγ as the target gene of miR-130-3p.

### Western blot

Protein extracts were resolved by 8%∼15% SDS–polyacrylamide gel, transferred to PVDF membranes, and probed with antibodies against α-SMA (ab32575, Abcam; 1:2,000), collagenI(ab34710, Abcam; 1:5,000), collagen III (ab7778, Abcam; 1:5,000), SOCS1 (#3950, Cell Signaling Technology; 1:1,000), PPARγ (sc-7196, Santa Cruz; 1:200), phospho-STAT6 (sc-11762, Santa Cruz; 1:500), and STAT6 (#9362, Cell Signaling Technology; 1:1,000), HDAC1 (#5356, Cell Signaling Technology; 1:1,000), and HDAC2 (#5113, Cell Signaling Technology; 1:1,000), GAPDH(HRP-60004, Proteintech; 1:10,000). Peroxidase-conjugated secondary antibody (CST) was used as the secondary antibody and the antigen-antibody reaction was visualized by enhanced chemiluminescence assay (ECL, Thermo). Western blot quantification was performed with ImageJ software, analysing the intensity of the grey scale images. The images have been cropped for presentation. Full size images are presented in Supplementary Fig. 7.

### qRT–PCR

qRT–PCR was performed with a LightCycler 480 instrument (Roche), using the SYBR Premix Ex Taq (TaKaRa, Japan) according to the manufacturer's instruction. The primer sequences are listed in Supplementary Table 3.

### Gene microarrays

mRNA microarray analysis was performed using 15 μg total RNA applied to Agilent_Whole Human Genome Microarray 4 × 44 K v2. The data were analysed using an Agilent Microarray Scanner (Agilent p/n G2565BA). The microarray data are available at the public databases (accession no. GSE59347).

### 3′UTR-luciferase reporter constructs

The wild-type or mutant 3′ UTRs of SOCS1 and PPARγ containing the predicted miR-142-5p or miR-130a-3p binding sites were synthesized (GeneArt, Lifetechnologies, Germany) and cloned into the pGL3.0-control vectors according to the manufacturer's instructions (Promega, Madison, WI). THP-1 cells(1 × 10^5^ cells per well) from American Type Culture Collection were seeded in 96-well plates. Cells were transfected with 10 pmol miR-142-5p mimics, miR-130a-3p mimics or scramble controls (Qiagen) and co-transfected with 0.2 μg per well wild-type SOCS1 3′ UTR-luc, mutant SOCS1 3′ UTR-luc, wild-type PPARγ 3′ UTR-luc or mutant PPARγ 3′ UTR-luc, respectively, using JetPEI transfection reagent (Polyplus transfection, Illkirch, France) according to the manufacturer's instructions. pRL-TK vectors (0.01 μg per well) were co-transfected as endogenous controls for luciferase activity. After the cells were transfected for 24 h, they were lysed, and luciferase activities were measured using a dual-luciferase assay kit (Promega). PPARγ complementary DNA carrying a wide-type 3′UTR or 3′UTR with a mutated seed sequence for miR-130a-3p (PPARγ-mut1) were cloned into pcDNA 3.1 for rescue experiments^58^.

### Promoter-luciferase reporter constructs

A fragment spanning from −1911 to +91 relative to the TSS of the human miR-142 genomic sequence and a fragment from −1891 to +91 relative to the TSS of the human miR-130a genomic sequence were produced from macrophages by PCR using the primers listed in Supplementary Table 3,respectively. These fragments were fused to pGL3-Basic vector (5'KpnI and 3' XhoI, Promega) to generate miR-142 (−1911/+91)-luc and miR-130a (−1891/+91)-luc, respectively. A series of nested deletions were generated using miR-142 (−1911/+91)-luc or miR-130a (−1891/+91)-luc as the template and the forward primers listed in Supplementary Table 3. The point mutations were introduced into the by QuikChange Site-Directed Mutagenesis Kit (Stratagene) using primers listed in Supplementary Table 3 according to manufacturer's instructions. The structures of all constructs were confirmed by DNA sequence analysis. MM6 cells were obtained from Lifetechnologies, China (https://www.lifetechnologies.com/cn/zh/home/technical- resources/cell-lines) and THP-1 cells were obtained from American Type Culture Collection. The cells were cultured in 10%FBS/RPMI 1640 medium and routinely tested for mycoplasma contamination using a single-step PCR method. For luciferase report experiments, MM6 or THP-1 cells grown in 96-well plates were transiently transfected with the indicated constructs (0.2 μg per well) and pRL-TK-Renilla (0.01 μg perwell) for 24 h using JetPEI transfection reagent according to the manufacturer's instructions, and then the cells were incubated with IL-4 (20 ng ml^−1^). After 16 h, the cells were harvested for the luciferase activity assay. To inhibit STAT6 activity, 100 μM leflunomide (Sigma) or 0.1 μM AS1517499 (Axon Medchem BV, Groningen, Netherlands) were added to culture media 30 min before the specified treatments. To inhibit SP1 activities, 0.1 μM mithramycin (Enzo Life Sciences, Shanghai, China) or 0.1 μM WP631(Sigma) was used.

### Electrophoretic mobility shift assays (EMSA)

Nuclear extracts were prepared using NE-PER nuclear extraction reagent (Pierce, Rockford, IL). DNA probes containing the STAT6 site of the miR-142 promoter or the SP1 site of the miR-130a promoter (Supplementary Table 3) were labelled at the 3'-end with biotin using a Biotin 3'End DNA Labelling Kit (Pierce), according to the manufacturer's instructions. The DNA binding reaction was performed using a Lightshift Chemiluminescent EMSA Kit (Pierce). A 50-fold molar excess of the unlabelled oligonucleotide was simultaneously added as a competitor with the labelled probe. To identify DNA binding proteins, nuclear extracts were incubated with 3 μg of antibody directed against STAT6 (sc-621X, Santa Cruz) Sp1 (sc-59X, Santa Cruz) or control IgG (AB-105-C, R&D Systems) at room temperature for 20 min before the addition of the labelled probe.

### Chromatin immunoprecipitation assay

ChIP was performed using a ChIP Assay kit (Millipore, Billerica, MA) according to the manufacturer's instructions. Briefly, 2 × 10^6^ (for AcH4, H3K4me3, H3K9me3 and H3K27me3) or 5 × 10^6^ (for STAT6, Sp1 RNA pol II and HDAC2) macrophages were stimulated with 20 ng ml^−1^ IL-4 for the indicated time, washed in PBS, and fixed in 1% formaldehyde for 10 min at room temperature. Fixed cells were harvested, lysed and sonicated for 10 cycles of 10 s on/20 s off and 50% AMPL with Sonics VCX130 (Sonics & Materials, Inc, Newtown). For AcH4 ChIP, sodium butyrate(20 mM, 19–137, Millipore) was added to all of the solutions to preserve histone acetylation. Antibodies directed against STAT6 (sc-621X; 5 μg per 1 mg total protein) and Sp1 (sc-59X; 5 μg per 1 mg total protein) were obtained from Santa Cruz, and antibodies directed against pol II (05–623; 10 μl per 1 mg total protein), AcH4(06–866; 10 μl per 1 mg total protein), H3K4me3 (07–473; 5 μl per 1 mg total protein), H3K9me3 (05–1242; 10 μl per 1 mg total protein), H3K27me3 (07–449; 5 μl per 1 mg total protein) and HDAC2 (17–10237; 5 μl per 1 mg total protein) were obtained from Millipore. The precipitated DNA was subjected to PCR amplification. The primer sequences used in ChIP assay are described in Supplementary Table 3.

### Fibrosis induction and treatment of mice

We induced liver fibrosis in B6.129S4-*Ccr2*^*tm1Ifc*^/J (The Jackson Laboratory) and wild-type C57BL/6J (8–10-week-old male) by gavage with CCL_4_ (Sigma, diluted 1:3 in corn oil) at a dose of 1 μl g^−1^ body weight every 5 days. We induced pulmonary fibrosis in the mice by intratracheal administration of 0.15 U bleomycin hydrochloride (Calbiochem) in 50 ml PBS. For adoptive monocyte transfer, monocytes were isolated from bone marrow of the femur and tibia by CD11b microbeads (Miltenyi Biotec, Germany). More than 90% purity of the isolated monocytes was confirmed by flow cytometry. *Ccr2*^*−/−*^ recipient mice were injected intravenously with 1 × 10^6^ monocytes or PBS. For LNA-modified oligonucleotides treatment, intravenous injection of LNA-modified miR-142-5p ASO, miR-130a-3p mimics, scramble control ASO or scramble control mimics (Exiqon) was performed 24 h after the initiation of CCL_4_ or 16 days after bleomycin challenge at a dose of 0.15 mg g^−1^ body weight and was repeated every 3 days thereafter. The mice were killed 6 weeks after the initiation of CCL_4_ challenge or 28 days after bleomycin challenge. All animal work was conducted in accordance with a protocol approved by the Institutional Animal Care and Use Committee at the Medical College of Sun Yat-Sen University.

### Histopathology and fibrosis

Paraffin-embedded samples were sectioned at 4-μm thickness. Antigen retrieval was performed in 0.01 M citrate buffer (pH 6.0) using a pressure cooker for 2 min, followed by treatment with 3% hydrogen peroxide for 5 min. Specimens were incubated with antibodies specific for α-SMA (MAB1420, R&D Systems; 1:100), overnight at 4 °C. Immunostaining was performed using DAB (Dako) according to the manufacturer's instructions. For negative control, isotype-matched antibodies were applied. Collagen fibres in liver were detected using a Sirius Red staining kit (Abcam) according to the manufacturer's instructions. Collagen fibres in lung tissues were detected by Masson's trichrome staining (collagen, blue; nuclei, dark red; cytoplasm, red/pink) as described previously^6^. We homogenized mouse whole lungs or livers in PBS and assessed acid-soluble collagen using a Sircol dye-binding assay kit (BioColor Ltd, Northern Ireland, UK) according to the manufacturer's instructions.

### Isolation of macrophages from lungs or livers of mice

Macrophages in the lungs or livers of mice were isolated as described previously^59^ with slight modifications. The lungs or livers were removed, minced and then incubated with collagenase I (Sigma) in DMEM supplemented with 10% FBS at 37 °C for 2 h. The cells were sequentially filtered through 500 μm mesh and 100 μm cell strainers. Red blood cells were lysed with RBC lysis buffer (500 ml dH_2_O, 4.15 g NH_4_Cl, 0.5 g KHCO_3_ and 0.019 g EDTA) and macrophages were isolated using CD11b microbeads (Miltenyi Biotec, Germany) according to the manufacturer's instructions.

### Human tissues

Liver cirrhosis tissues were obtained from 39 patients undergoing transplantation for hepatitis B-induced liver failure, and normal liver tissues were obtained from 24 patients undergoing surgery for hepatic haemangioma. BAL fluid samples were obtained by bronchoscopy from seven normal volunteers and nine patients diagnosed with IPF from Sun Yat-Sen Memorial Hospital and the Third Affiliated Hospital of Sun Yat-sen University. All samples were collected with the informed consent of the patients, and all related procedures were performed with the approval of the Internal Review and Ethics Boards of the indicated hospitals.

### Fluorescence *in situ* hybridization (FISH) and CD68 staining

The combination of immunofluorescence and FISH experiments was performed as previously described with slight modifications^60^. Briefly, the paraffin embedded sections were deparaffinized and incubated with primary mouse anti-CD68 monoclonal antibody(sc-17832, Santa Cruz; 1:200). Alexa Fluor 555-conjugated donkey anti-mouse IgG (Invitrogen, CA, USA) was used. The sections were washed twice with PBS and dehydrated by ethanol. The sections were rehydrated in 50% formamide in 2 × SSC for 5 min and prehybridized in 2 × SSC for 1 h at 42 °C. Then, the sections were hybridized with 20 nM 5' digoxigenin-labelled LNA control probe and miR-142-5p or miR-130-3p probe (Exiqon) overnight at 42 °C, then rinsed twice with 5 × SSC at room temperature, washed three times in 2 × SSC/50% formamide at hybridization temperature for 20 min and washed four times in PBS with 0.1% Tween 20 (PBST). Sections were blocked for endogenous peroxidase activity (30 min in 3% H_2_O_2_ in PBST) and incubated with blocking buffer (0.5% blocking reagent with 10% serum) for 1 h and with anti-digoxigenin-FITC antibody (Roche, 1:10,000) overnight at 4 °C. Sections were washed twice in PBST for 5 min and counter stained with DAPI (Invitrogen). Cells stained with the indicated probe were calculated per field of view and at least 10 fields per section were evaluated at 400 × magnification.

### Isolation of macrophages from BAL fluid

Bronchoscopy is performed as previously described^61^. CD14^+^ monocytes/macrophages were isolated from BAL fluid cells by a magnetic-activated cell sorting (MACS) using direct CD14 Isolation Kit (Miltenyi) according to the manufacturer's instructions (approximate 0.8–5 × 10^4^ cells per sample).

## Additional information

**How to cite this article:** Su, S. *et al*. miR-142-5p and miR-130a-3p are regulated by IL-4 and IL-13 and control profibrogenic macrophage program. *Nat. Commun.* 6:8523 doi: 10.1038/ncomms9523 (2015).

## Supplementary Material

Supplementary Information Supplementary Figures 1-7 and Supplementary Tables 1-3
