# Supplementary material for: miR-142-5p and miR-130a-3p are regulated by IL-4 and IL-13 and control profibrogenic macrophage program
Source: Nat Commun. 2015 Oct 5;6:8523. doi: 10.1038/ncomms9523 (PMC4600756; doi:10.1038/ncomms9523)
Supplement: Supplementary Information — Supplementary Figures 1-7 and Supplementary Tables 1-3 [file ncomms9523-s1.pdf]

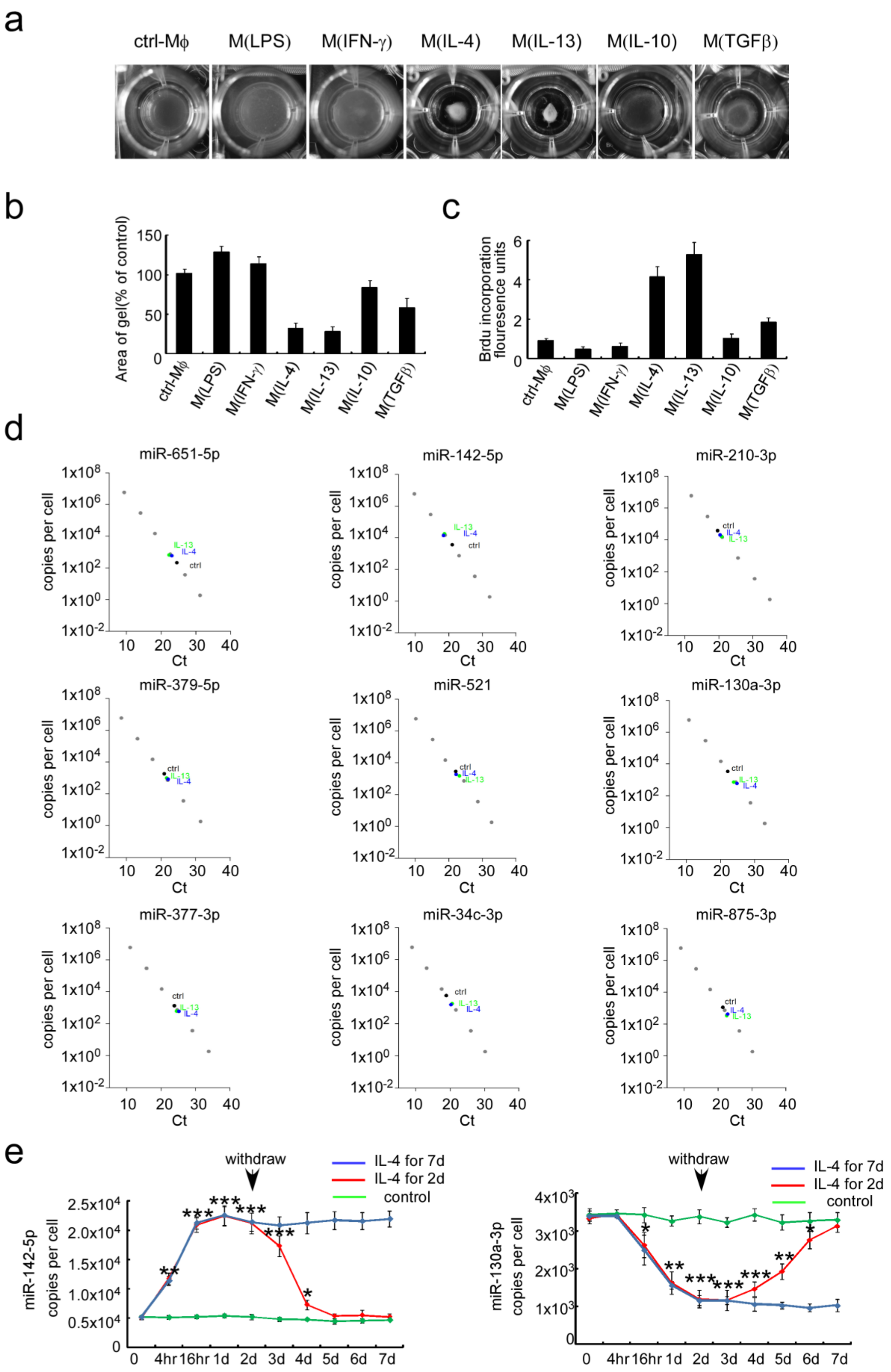

Supplementary Figure 1. Profibrogenic macrophages exhibit unique miRNA expression profiles.(a-c) The macrophages were stimulated with or without LPS, IFN-γ, IL-4, IL-13, IL-10 or TGF-β for 12 hr and co-cultured with primary human fibroblasts for another 48 hr.(**a**) Fibroblast contractility in three-dimensional collagen matrices. The representative images are shown.(**b**) Gel area treated as in (**a**) were

quantified using ImageJ software. (mean ± s.e.m., n=4 independent experiments).(**c**) The proliferation of fibroblasts was determined as by BrdU incorporation assay (mean ± s.e.m., n=4 independent experiments).(**d**) Estimated copy numbers of miRNAs per macrophages. Equivalent molecules per cell were calculated based on the assumption that total RNA per cell is 20 pg. Black dots, blue ones and green ones indicate average molecules per macrophages treated by PBS, IL-4 and IL-13, respectively (n=4). (**e**) Macrophages were treated with IL-4 for 2 days. Afterward, the media were replaced and macrophages were treated with (IL4 for 7d) or without IL-4 (IL4 for 2d) for another 5 days. The expression of miR-142-5p and miR-130a-3p were examined by qRT-PCR at the indicated time points (mean ± s.e.m., n=3 independent experiments; *, $p<0.05$; **, $p<0.01$, ***, $p<0.001$ compared to control cells by two-tailed Student's t-tests)

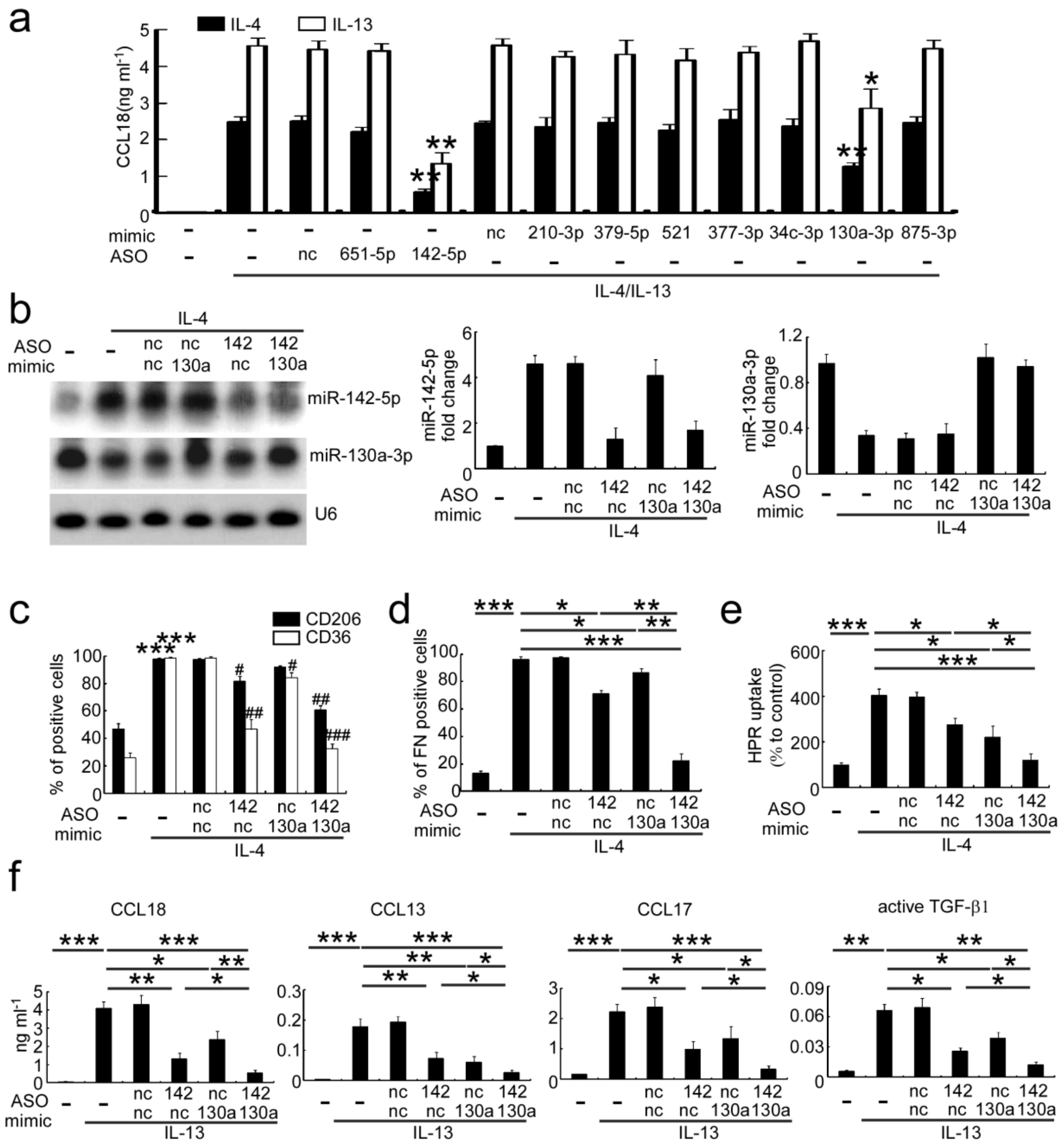

Supplementary Figure 2. miR-142-5p and 130a-3p control M2 polarization(**a**) Macrophages were transduced with indicated oligonucleotides using lentiviral vectors. After 24 hr, the cells were treated with IL-4 or IL-13 for 48 hr. CCL18 levels in the media were measured by ELISA (mean ± s.e.m, n=3 independent experiments; *, p<0.05; **, p<0.01 vs. IL-4/IL-13 activated macrophages transduced with control oligonucleotides. p values were obtained using two-tailed Student's t-tests).(**b**) Macrophages were transduced with negative control (nc) or miR-142-5p ASO or miR-130a-3p mimics or both using lentiviral vectors. After 24 hr, the cells were treated with IL-4 for 24 hr. The expression of miR-142-5p and miR-130a-3p of indicated cells were measured by Northern blot (left panels) and qRT-PCR (right panels, n=3).(**c**) Percentages of CD206 or CD36 positive macrophages treated as in **Fig. 2b** were determined as by flow-cytometry analysis. (mean ± s.e.m., n=4 independent experiments; ***, p<0.001 compared to control cells. #, p<0.05; ##, p<0.01; ###, p<0.001 compared to macrophages treated with IL-4 alone. p values

were obtained using two-tailed Student's t-test).(**d**) Percentages of fibronectin positive macrophages treated as in **Fig. 2c** were quantitated by Image J (mean ± s.e.m, n=3 independent experiments; *, p<0.05; **, p<0.01; ***, p<0.001. p values were obtained using two-tailed Student's t-test).(**e**) Macrophages were transduced with negative control (nc) or miR-142-5p ASO or miR-130a-3p mimics or both using lentiviral vectors. After 24 hr, the cells were treated with IL-4 for 48 hr and pulsed with 1 mg ml^{-1} HRP for 2 hr. Measurements are expressed as the mean percentage of HRP uptake in untreated controls (mean ± s.e.m., n=4 independent experiments; *, p<0.05; ***, p<0.001. p values were obtained using two-tailed Student's t-test).(**f**) Macrophages were transduced with negative control (nc) or miR-142-5p ASO or miR-130a-3p mimics or both using lentiviral vectors. After 24 hr, the cells were treated with IL-13 for 48 hr. Cytokine levels in the media of macrophages were measured by ELISA (mean ± s.e.m., n=4 independent experiments; *, p<0.05; **, p<0.01; ***, p<0.001. p values were obtained using a two-tailed Student's t test).

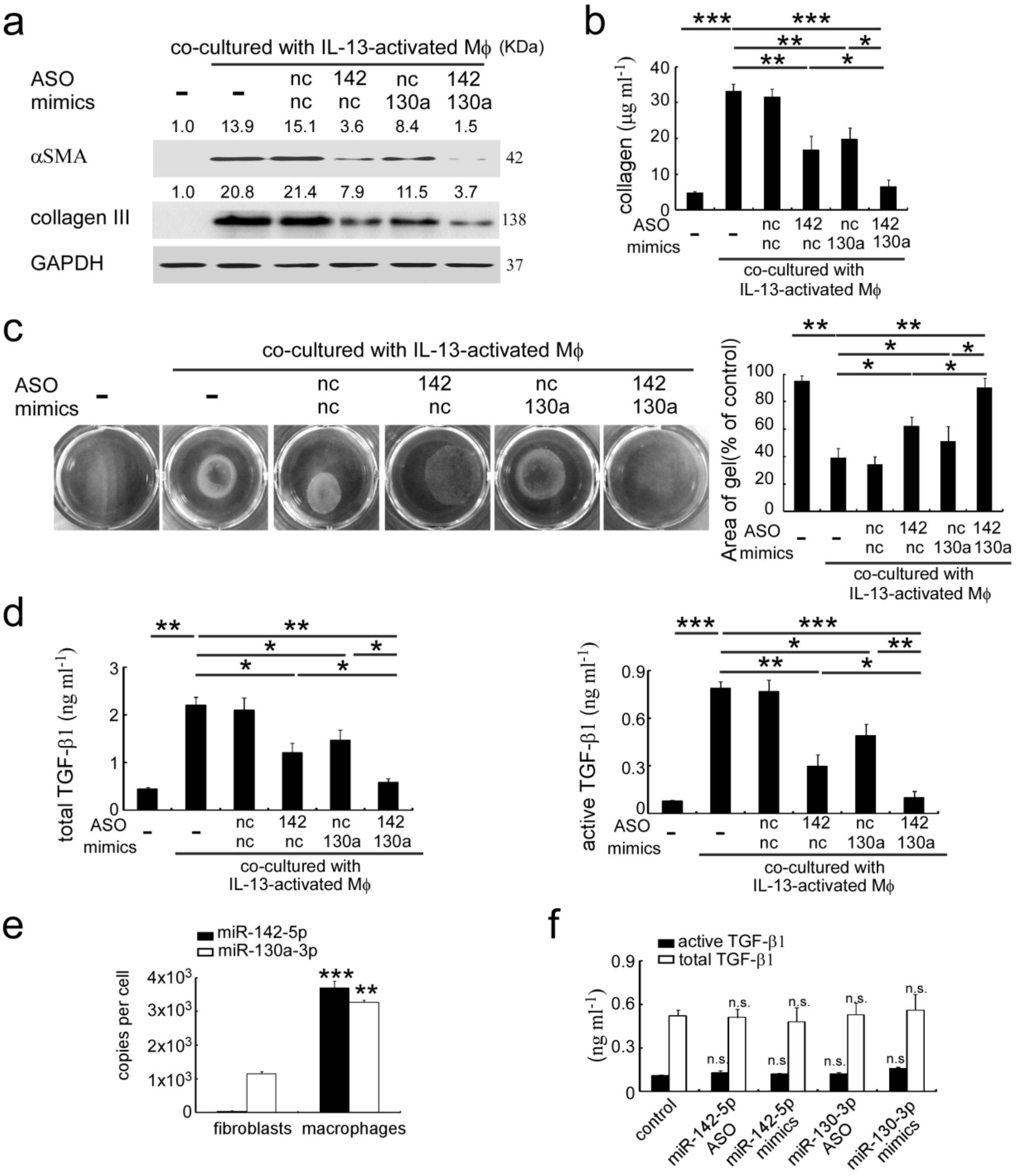

Supplementary Figure 3. miR-142-5p and 130a-3p regulate Mφ profibrogenesis

(**a**-**d**) Macrophages were transduced with control or miR-142-5p ASO or miR-130a-3p mimics or both. After 24 hr, the macrophages were stimulated with IL-13 for 12 hr and co-cultured with primary human fibroblasts for another 48 hr.(**a**) Representative images of western blot for α-SMA and collagen III in fibroblasts. The numbers above the blots present the intensity ratio of indicated protein/GAPDH analyzed by ImageJ (n=2).(**b**) Extracellular acid-soluble collagen production of fibroblasts was measured by the Sircol assay. (mean ± s.e.m., n=4 independent experiments; **, $p<0.01$; ***, $p<0.001$. p values were obtained using two-tailed Student's t-test).(**c**) Fibroblast contractility in three-dimensional collagen matrix (mean ± s.e.m., n=4 independent experiments; *, $p<0.01$; **, $p<0.001$. p values were obtained using two-tailed Student's t-tests).(**d**) Total TGF-β1 (acid-treated) and active TGF-β1 (not acid-treated) in the media of macrophage/fibroblast co-culture system

(mean ± s.e.m., n=4 independent experiments; *, $p<0.05$; **, $p<0.01$; ***, $p<0.001$. p values were obtained using two-tailed Student's t-test). (**e**) Absolute quantification by qRT-PCR for the miR-142-5p and miR-130a-3p expression in primary human fibroblasts and macrophages (mean ± s.e.m., n=4; **, $p<0.01$; ***, $p<0.001$, compared with fibroblasts by two-tailed Student's t-test). (**f**) Fibroblasts were transduced with ASO or mimics of miR-142-5p or miR-130a-3p mimics using lentiviral vectors. After 48 hr, levels of active or total TGF-β1 in the media were evaluated by ELISA. (mean ±s.e.m., n=3, n.s., not statistically significant compared to control.)

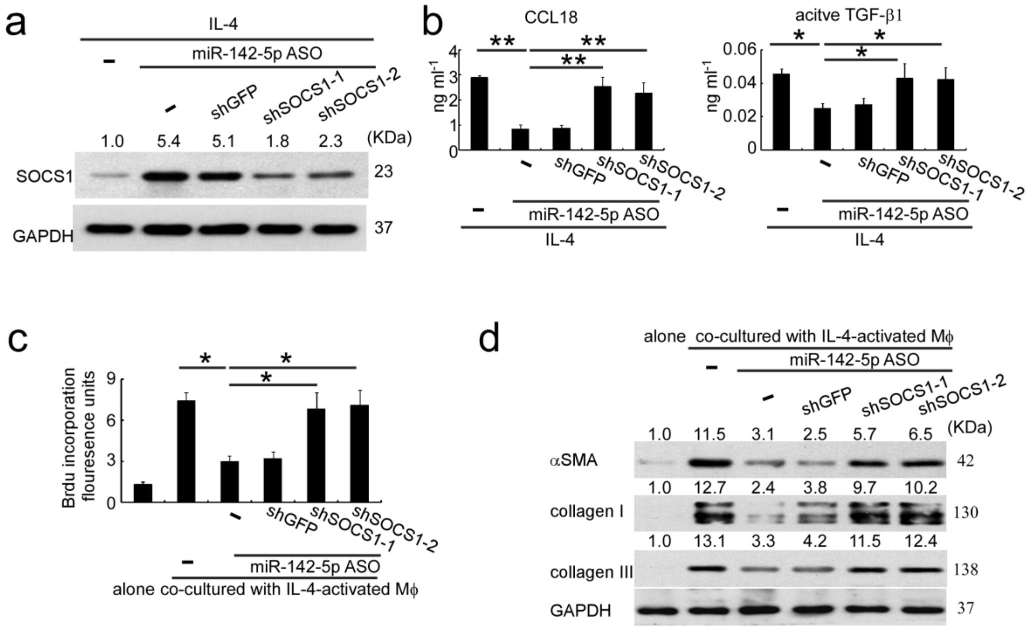

Supplementary Figure 4. miR-142-5p regulates profibrogenesis by targeting SOCS1 (**a-d**) Macrophages were transduced with miR-142-5p ASO or co-transduced with shRNAs targeting GFP or SOCS1. After 24 hr, the macrophages were stimulated with IL-4 for 12 hr and co-cultured with primary human fibroblasts for another 48 hr.(**a**) Representative images of western blot for SOCS1 in macrophages (n=2). The numbers above the blots present the intensity ratio of SOCS1/GAPDH analyzed by ImageJ.(**b**) Cytokine levels in the media of macrophages (mean ± s.e.m., n=4; *, $p<0.05$; **, $p<0.01$ by two-tailed Student's t-test).(**c**) The proliferation of fibroblasts was determined by BrdU incorporation assay (mean ± s.e.m., n=4; *, $p<0.05$ by two-tailed Student's t-test).(**d**) Representative images of western blot for α-SMA, collagen I and collagen III in fibroblasts (n=2). The numbers above the blots present the intensity ratio of indicated protein/GAPDH analyzed by ImageJ.

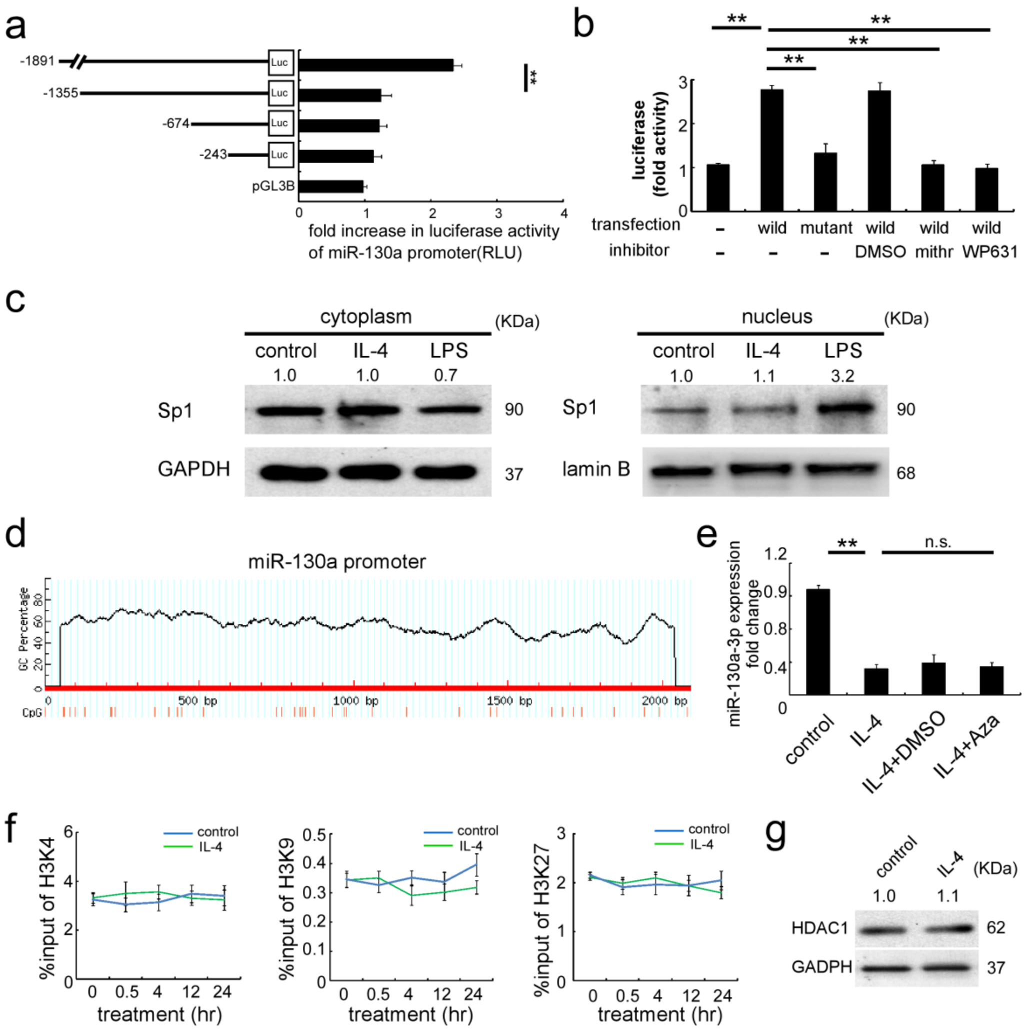

Supplementary Figure 5. IL-4 downregulates miR-130a-3p by histone deacetylation (**a**) MM6 cells were transiently transfected with whole length or several deletion miR130a-luc constructs and pRL-TK-Renilla and harvested for the luciferase activity assay (mean ± s.e.m., n=3 independent experiments; **, p<0.01 using two-tailed Student's t-tests)(**b**) MM6 cells were transfected with a full-length or mutated reporter construct of miR130a and then exposed to DMSO or Sp1 inhibitors (mithramycin, mithr or WP631). Cells were harvested for the luciferase activity assay(mean ± s.e.m., n=3 independent experiments; **, p<0.01 using two-tailed Student's t-tests)(**c**) Primary human macrophages were incubated with or without IL-4 or LPS for 30 mins, Sp1 accumulations in cytoplasm and nucleus were determined by immunoblotting (n=2). The numbers above the blots present the intensity ratio of Sp1/GAPDH or laminB analyzed by ImageJ.(**d**) Schematic representation of the promoter region of miR-130a. CpG sites are shown as vertical bars.(**e**) Macrophages were stimulated with 20ng ml^{-1} IL-4 with or without pretreatment of DMSO or 5-Aza-dC (Aza).miR-130a expression was determined by qRT-PCR 24 hr afterward (mean ± s.e.m., n=3 independent experiments; **, p<0.01; n.s. not significant using two-tailed Student's t-tests).(**f**) Macrophages were stimulated with 20ng ml^{-1} IL-4 for indicated time and kinetics of H3K4me3, H3K9me3 or H3K27me3 on the miR-130a promoter were analyzed by ChIP. Results are presented as enrichment (percentage of

input DNA) of H3K4me3, H3K9me3 or H3K27me3 promoter occupancy(mean ± s.e.m., n=3 independent experiments).(**g**) Macrophages were stimulated with IL-4 for 48 hr and the protein level of HADC1 was determined by western blot(n=3).

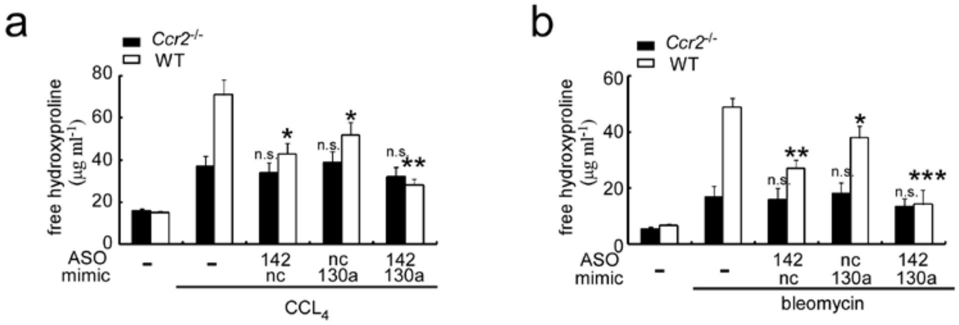

Supplementary Figure 6. LNA-modified miRNA treatments work by blocking Mφ profibrogenic function (**a**) Wild-type mice or *Ccr2*$^{-/-}$ mice were intravenously injected with LNA-modified miR-142-5p ASO, miR-130a-3p mimic or both every 3 days after CCL_4 challenge. Mice were sacrificed and liver hydroxyproline was measured after 6 weeks (mean ± s.e.m., n=8 mice/group; n.s., not statistically significant, *, $p<0.05$, **, $p<0.01$ by two-tailed Student's t-test, compared to mice challenged with CCL_4 without treatment).(**b**) Wild-type mice or *Ccr2*$^{-/-}$ mice were intravenously injected with LNA-modified miR-142-5p ASO, miR-130a-3p mimics or both 16 days after intratracheal administration of bleomycin and repeated every 3 days. Mice were sacrificed and lung hydroxyproline was measured 28 days following bleomycin challenge (mean ± s.e.m., n=8 mice/group; n.s., not statistically significant, *, $p<0.05$, **, $p<0.01$ ***, $p<0.001$ by two-tailed Student's t-test, compared to mice challenged with bleomycin without treatment).

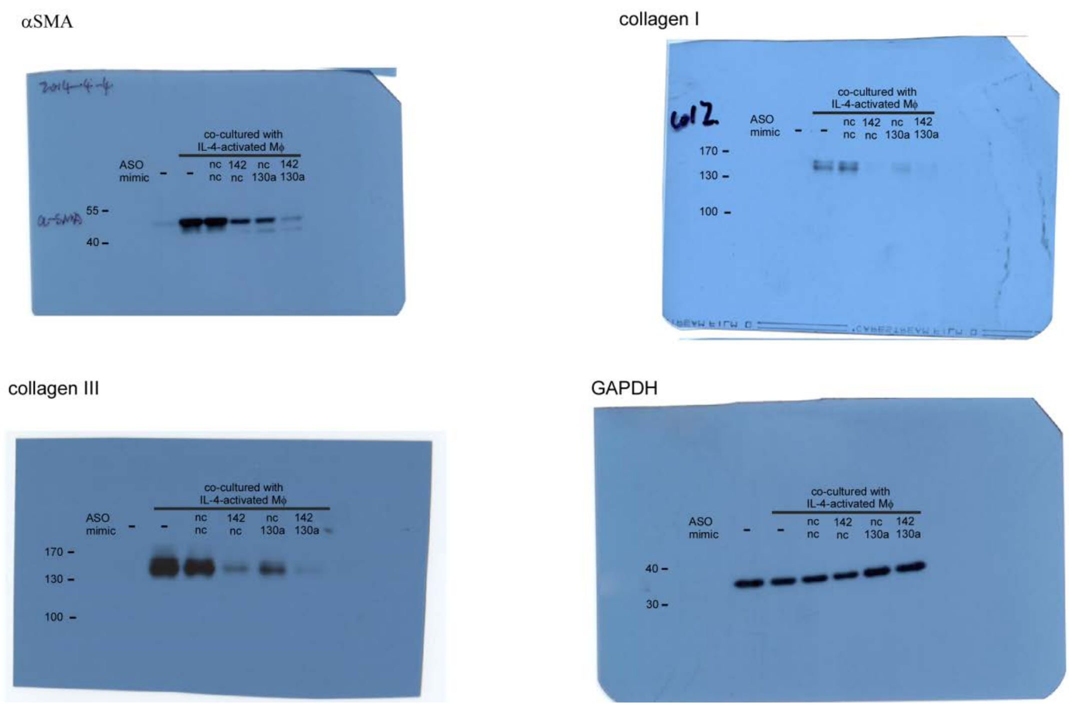

Supplementary Figure 7a. Uncropped blots corresponding to Figure 3d

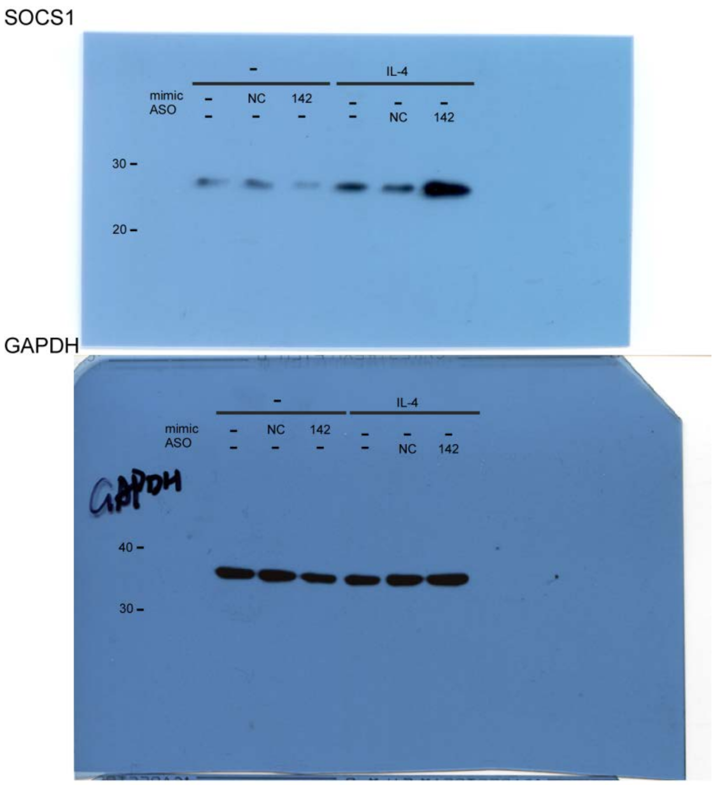

Supplementary Figure 7b. Uncropped blots corresponding to Figure 4d

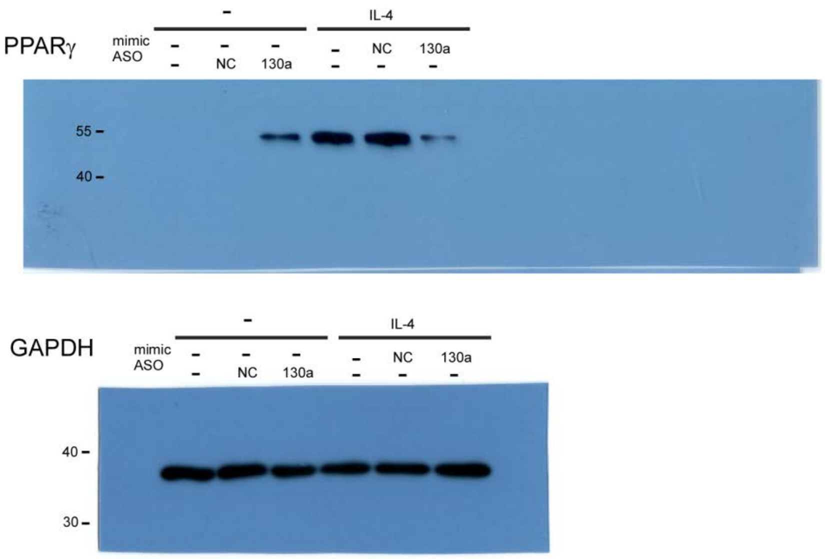

Supplementary Figure7c. Uncropped blots corresponding to Figure 4f

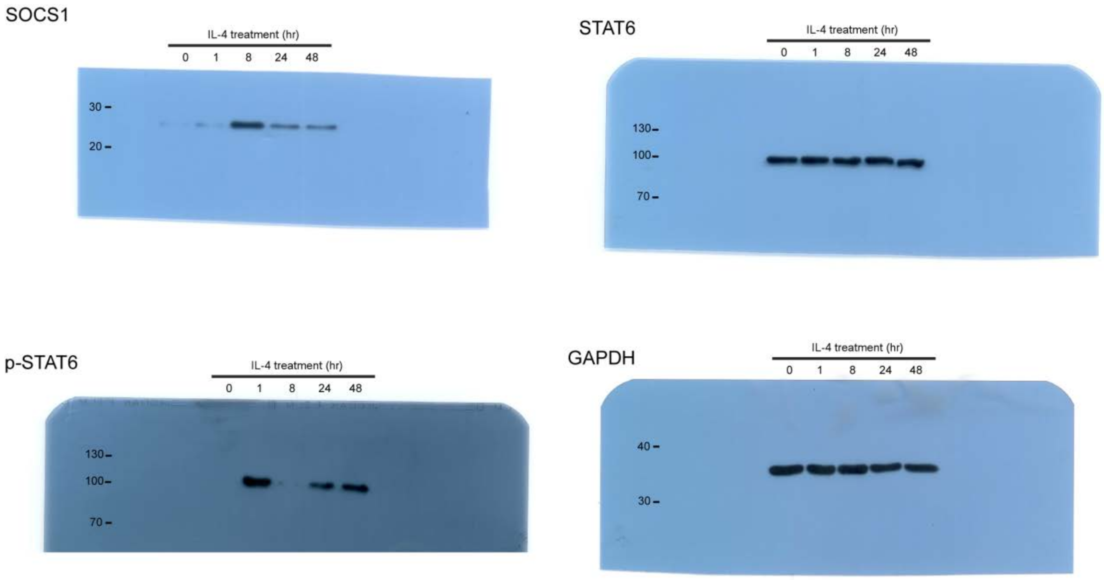

Supplementary Figure 7d. Uncropped blots corresponding to Figure 4g.

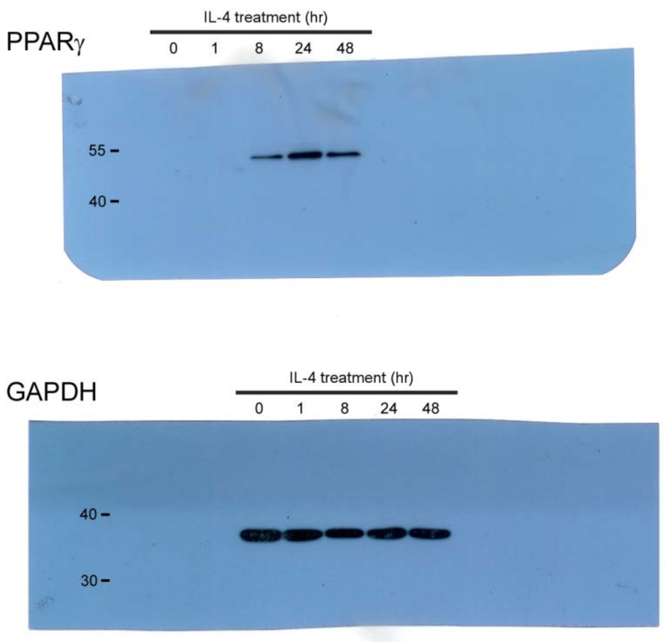

Supplementary Figure 7e. Uncropped blots corresponding to Figure 4h

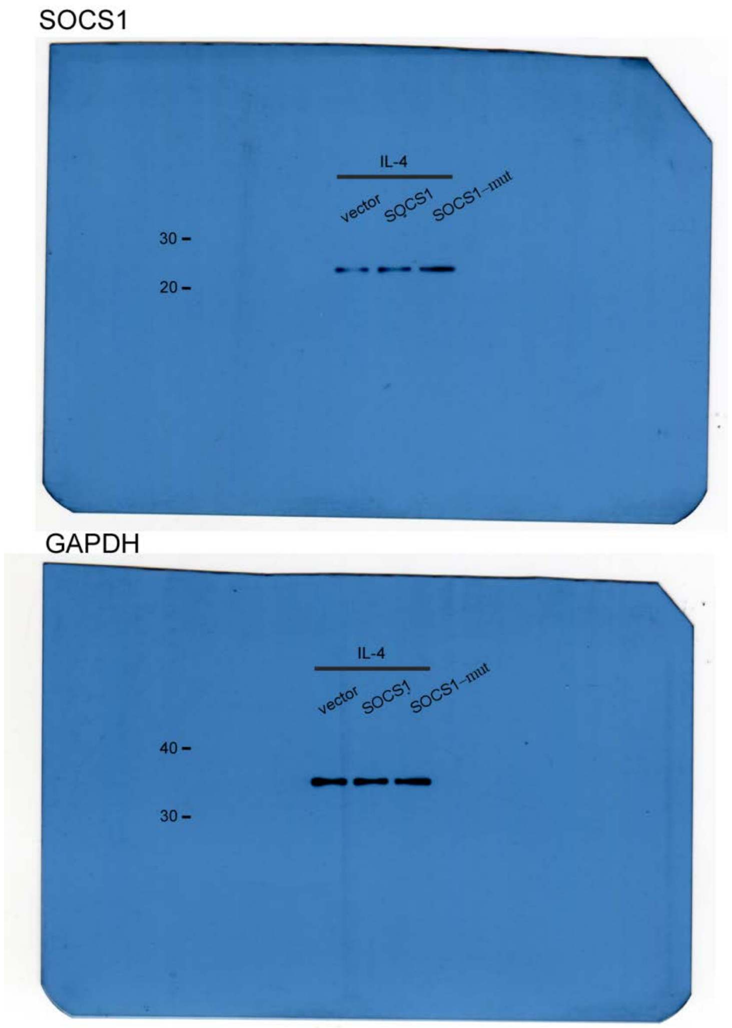

Supplementary Figure 7f. Uncropped blots corresponding to Figure 5a

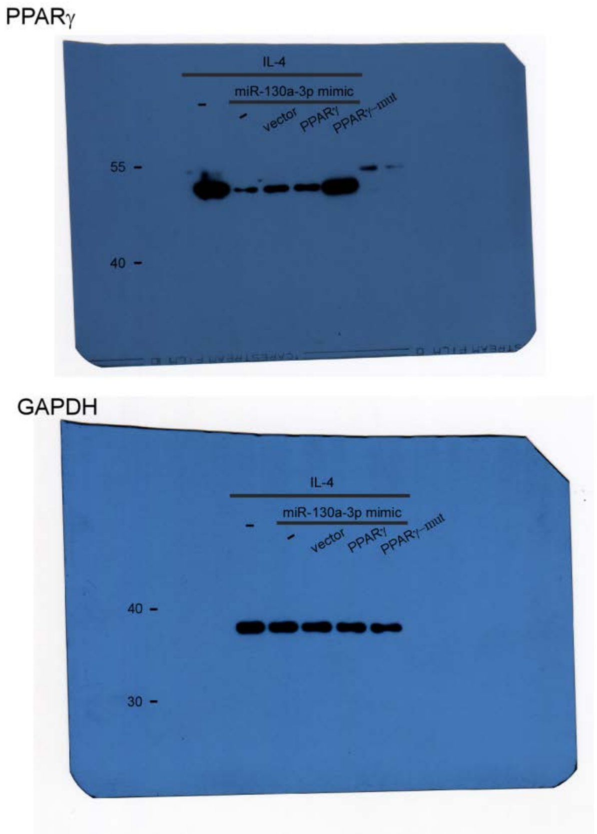

Supplementary Figure 7g. Uncropped blots corresponding to Figure 5d

the STAT6 binding site in miR-142 promoter

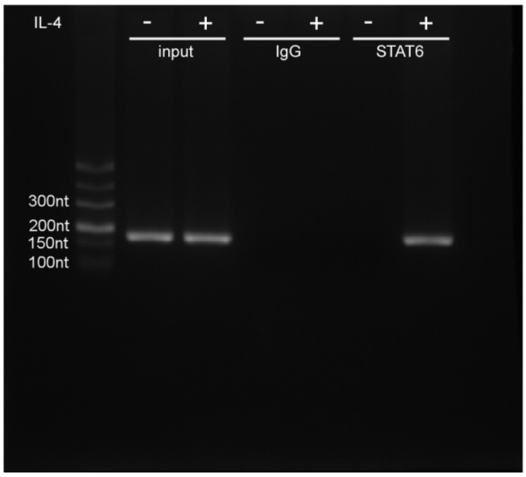

Supplementary Figure 7h. Uncropped blots corresponding to Figure 7d

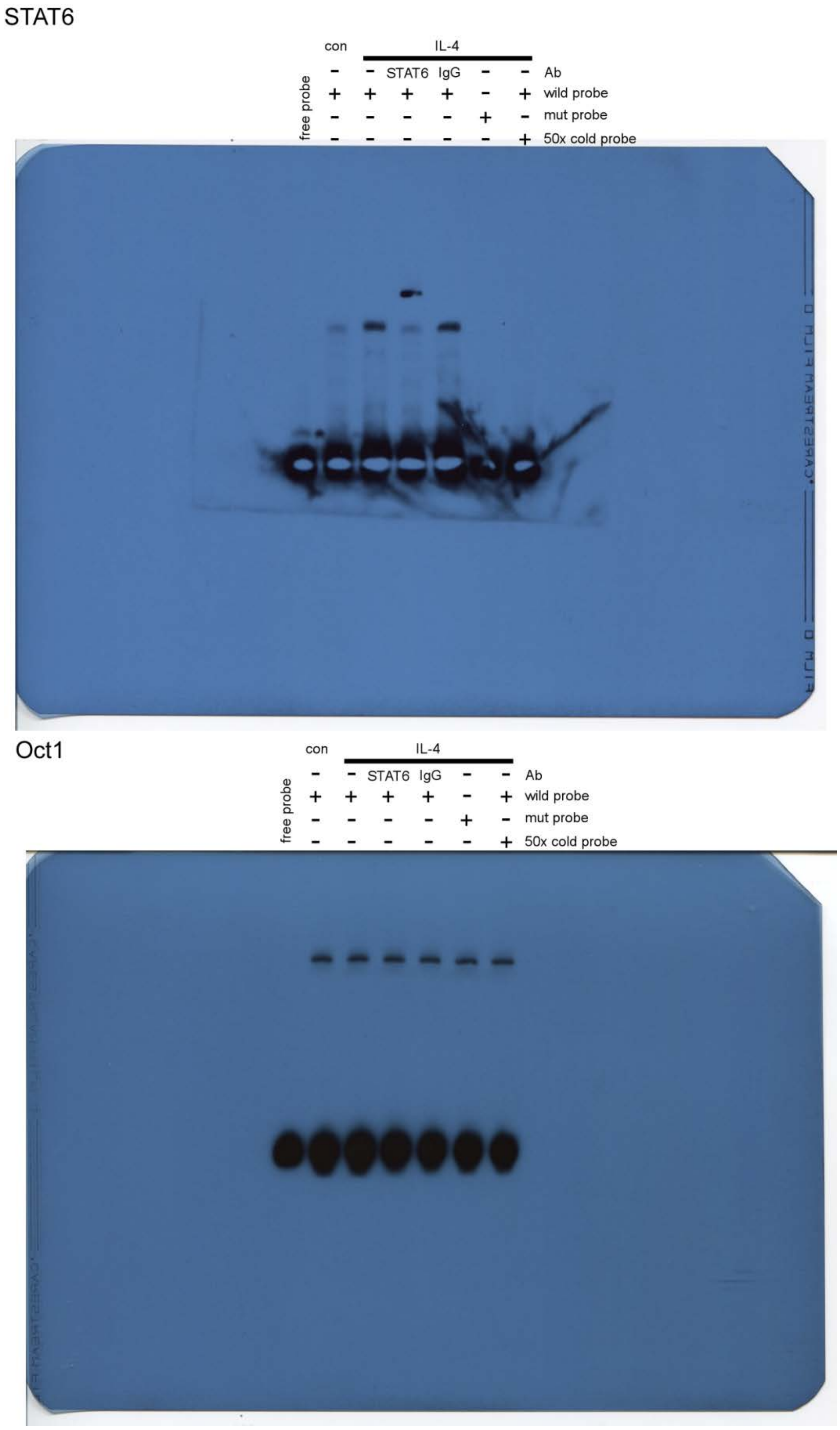

Supplementary Figure 7i. Uncropped blots corresponding to Figure 7e

the Sp1 binding site in miR-130a promoter

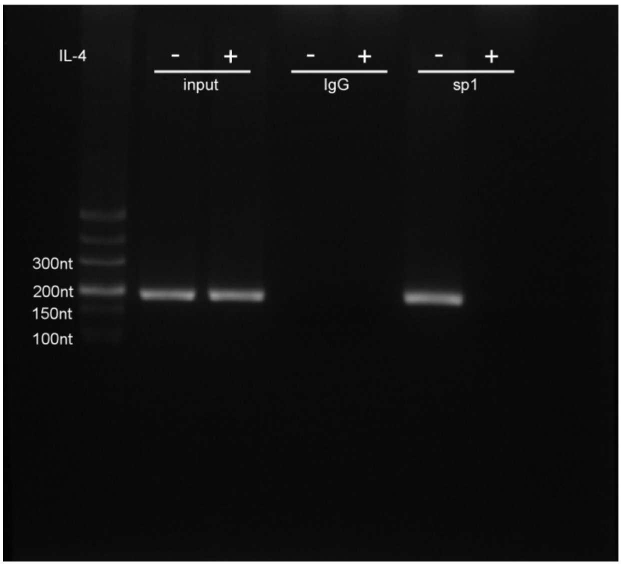

Supplementary Figure 7j. Uncropped blots corresponding to Figure 8b

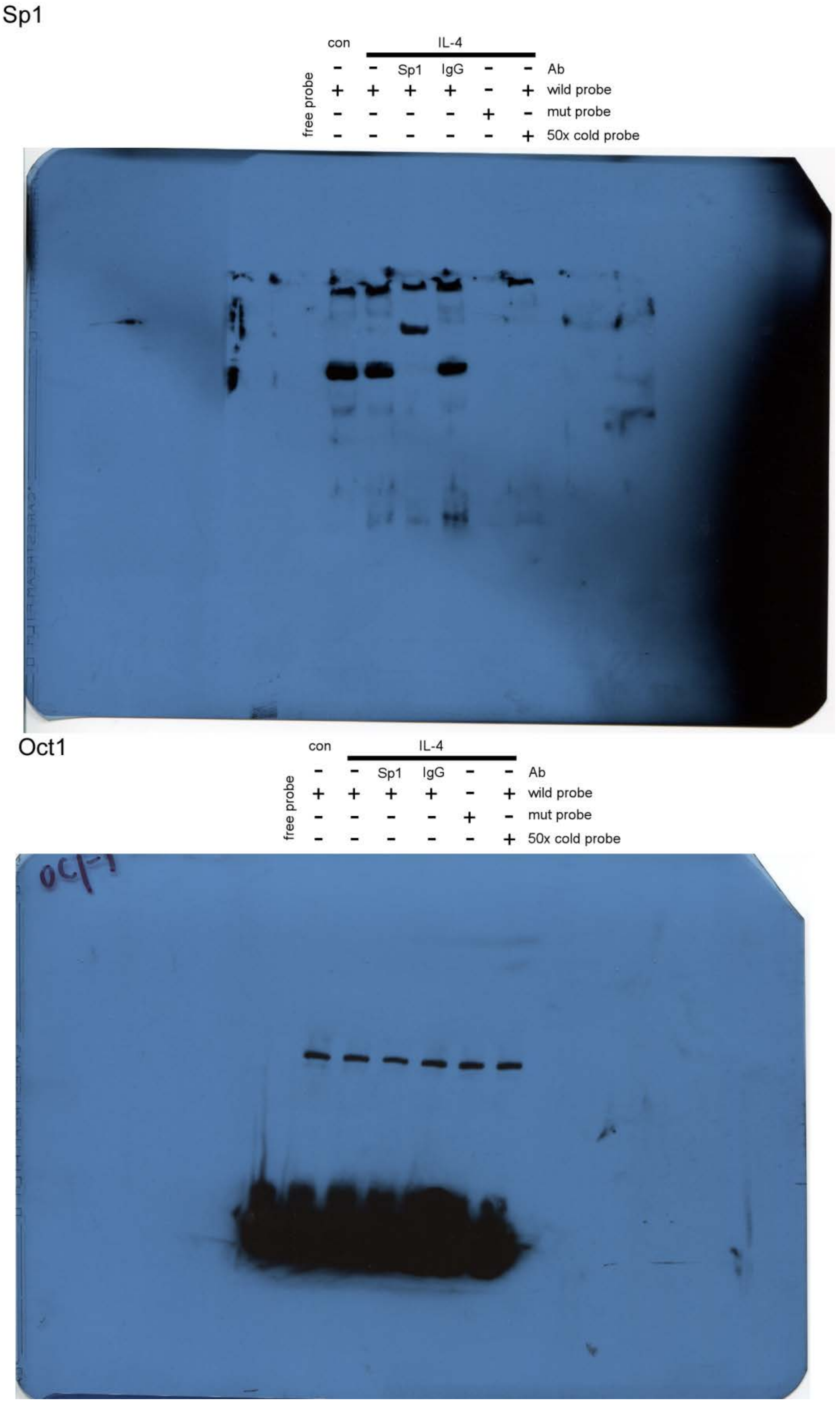

Supplementary Figure 7k. Uncropped blots corresponding to Figure 8c

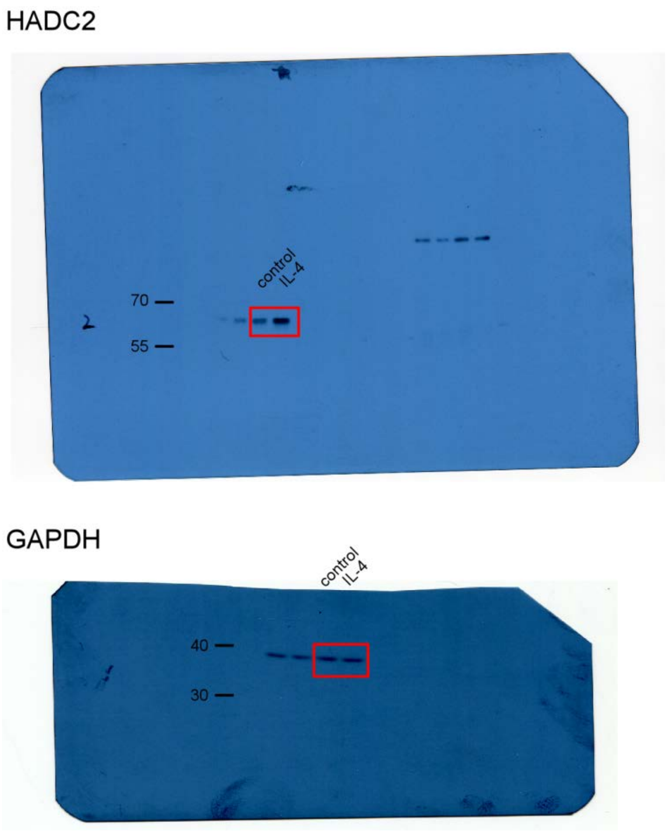

Supplementary Figure 7l. Uncropped blots corresponding to Figure 8h

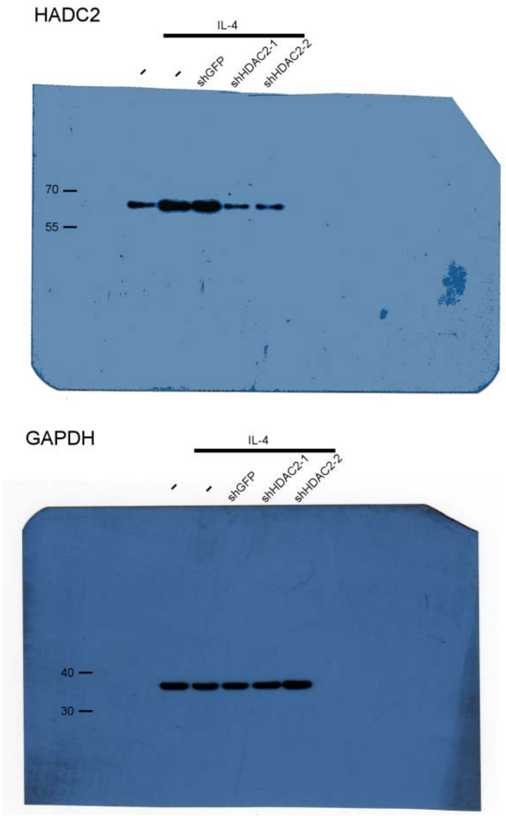

Supplementary Figure 7m. Uncropped blots corresponding to Figure 8j

HADC2

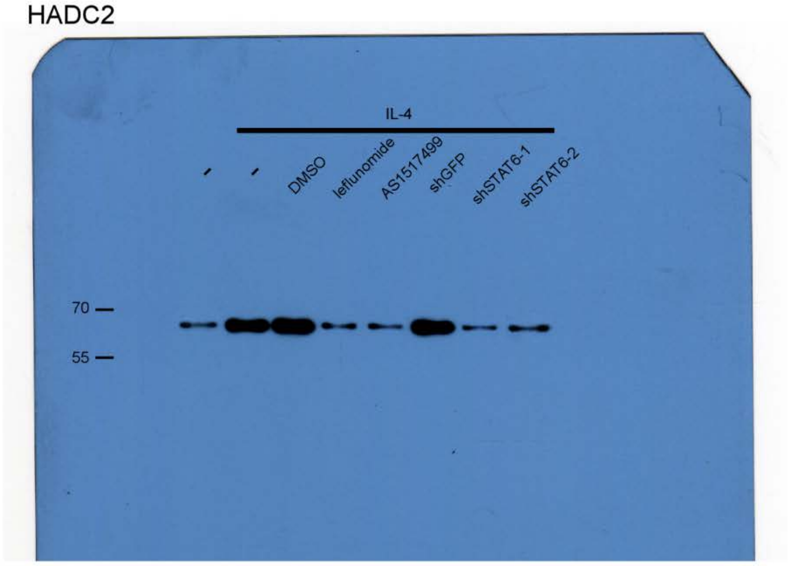

GAPDH

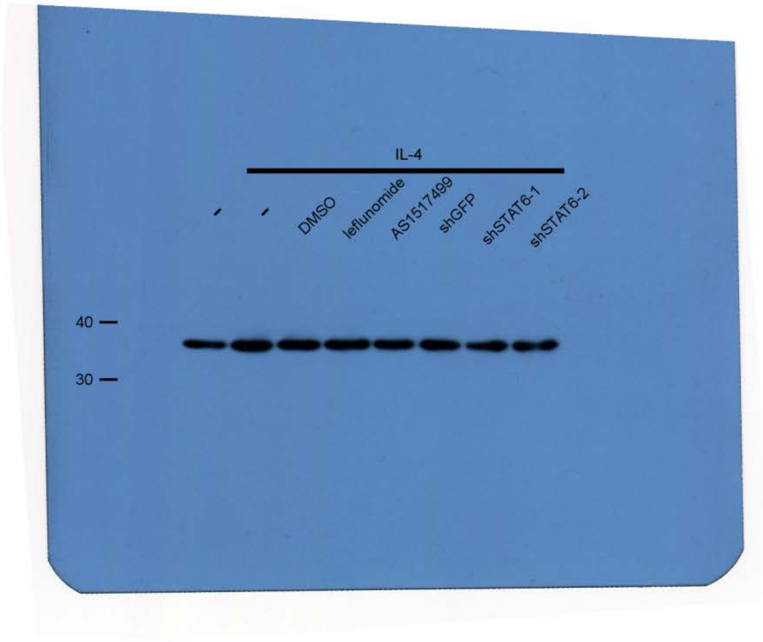

Supplementary Figure 7n. Uncropped blots corresponding to Figure 8l

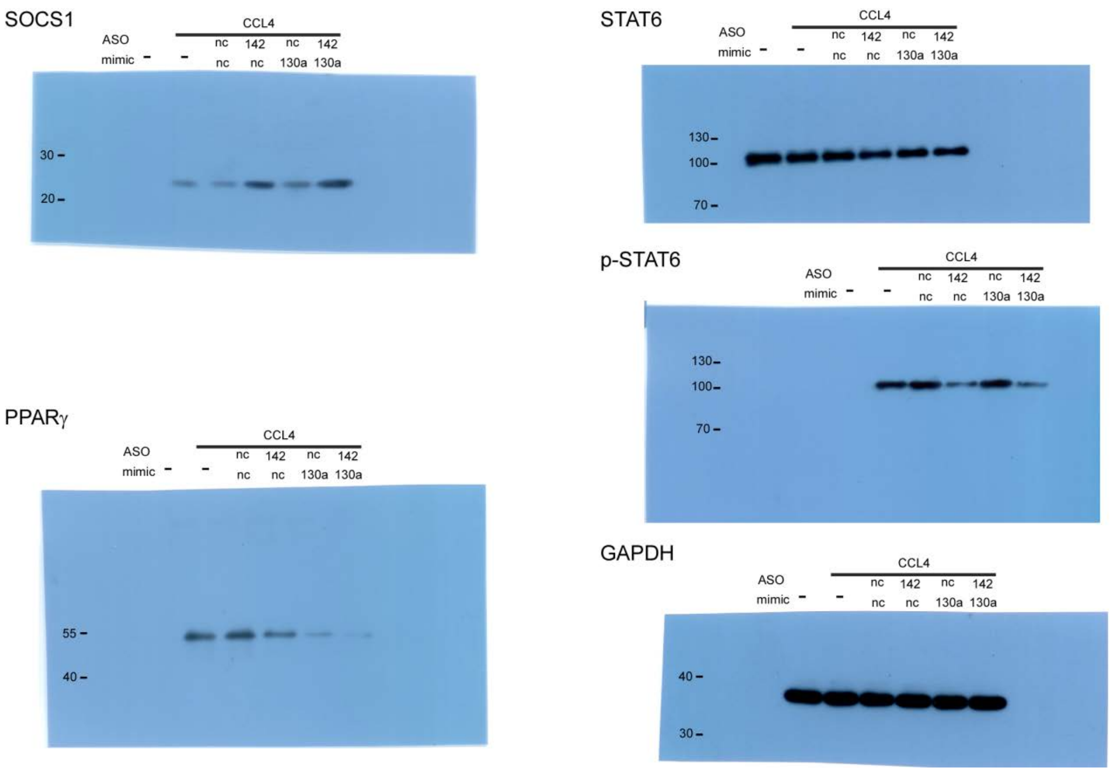

Supplementary Figure 7o. Uncropped blots corresponding to Figure 9c

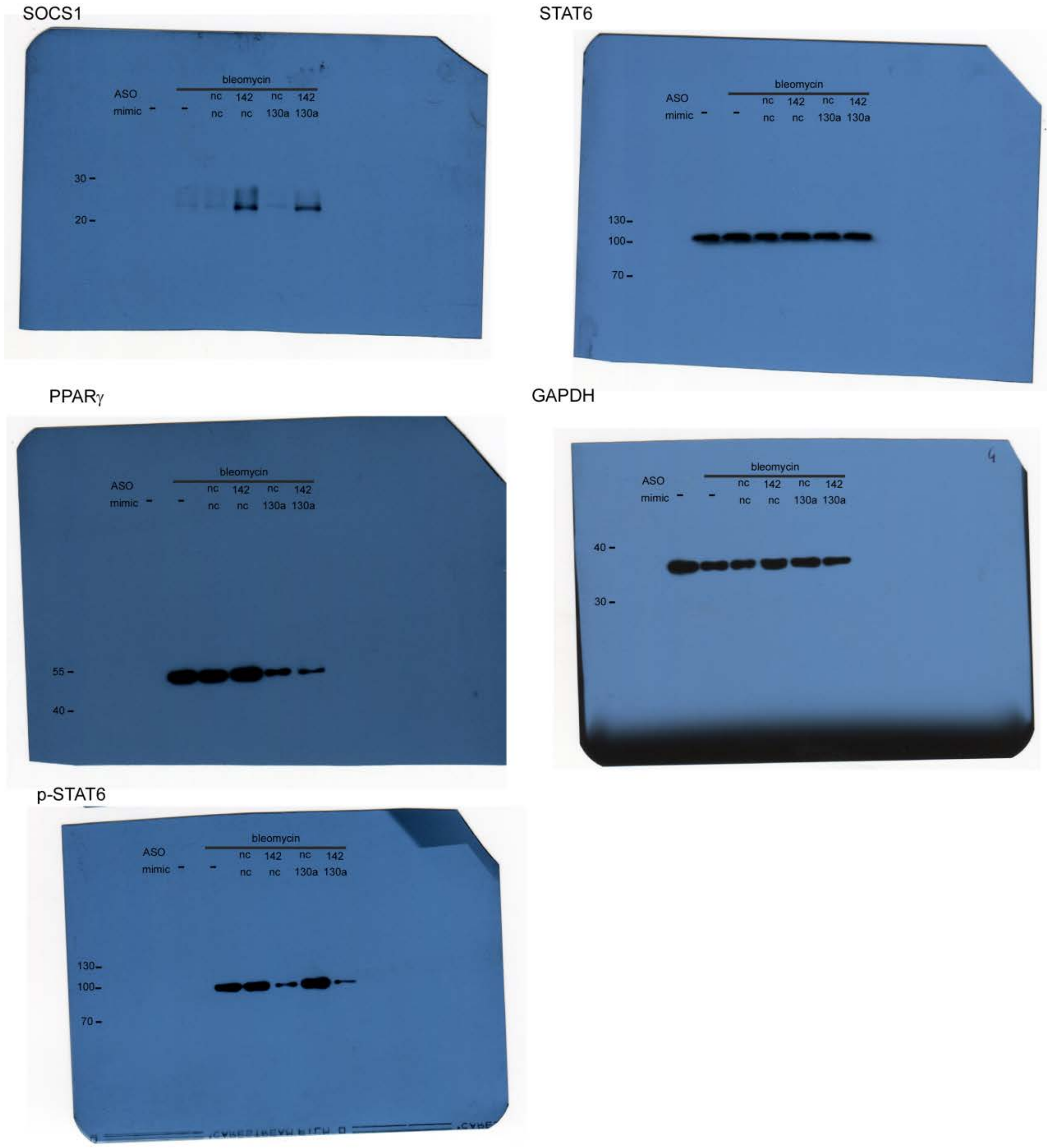

Supplementary Figure 7p. Uncropped blots corresponding to Figure 10f

CCL18

CI (% effect)		miR-130a-3p mimic (MOI)			
		0	2.5	5	10
miR-142-5p ASO (MOI)	0	1.91 (0)	1.05 (9)	1.02 (22)	1.05 (43)
	2.5	1.07 (31)	0.33 (75)	0.23 (88)	0.22 (93)
	5	0.92 (58)	0.22 (91)	0.17 (95)	0.19 (96)
	10	1.07 (72)	0.32 (93)	0.08 (99)	0.16 (98)

CCL13

CI (% effect)		miR-130a-3p mimic (MOI)			
		0	2.5	5	10
miR-142-5p ASO (MOI)	0	1.91 (0)	0.95 (17)	0.89 (34)	1.04 (51)
	2.5	1.08 (16)	0.42 (50)	0.33 (71)	0.42 (78)
	5	0.97 (38)	0.32 (73)	0.22 (87)	0.21 (93)
	10	0.88 (72)	0.23 (90)	0.26 (91)	0.18 (96)

CCL17

CI (% effect)		miR-130a-3p mimic (MOI)			
		0	2.5	5	10
miR-142-5p ASO (MOI)	0	1.92 (0)	0.95 (9)	0.89 (21)	1.05 (33)
	2.5	0.97 (22)	0.75 (41)	0.57 (59)	0.46 (75)
	5	1.05 (43)	0.50 (75)	0.41 (83)	0.35 (89)
	10	1.20 (65)	0.49 (89)	0.26 (96)	0.38 (94)

active TGF-β1

CI (% effect)		miR-130a-3p mimic (MOI)			
		0	2.5	5	10
miR-142-5p ASO (MOI)	0	1.91 (0)	1.09 (7)	0.93 (24)	1.10 (37)
	2.5	1.16 (10)	0.34 (58)	0.39 (66)	0.58 (69)
	5	0.94 (35)	0.44 (64)	0.38 (76)	0.45 (81)
	10	1.16 (43)	0.51 (75)	0.43 (83)	0.42 (88)

Supplementary Table 1. miR-142-5p and 130a-3p synergistically control M2 polarization. Tables showing combination index (CI) values and % inhibition of cytokine production (relative to untransduced cells) for IL-4 stimulated macrophages transduced with miR-142-5p ASO or/and miR-130a-3p mimics at various multiplicity of infection (MOI). Reported CI values represent the average of four replicate experiments. CI values smaller than 0.5 indicate strong synergy.

collagen production

CI (% effect)		miR-130a-3p mimic (MOI)			
		0	2.5	5	10
miR-142-5p ASO (MOI)	0	1.91 (0)	1.56 (17)	0.87 (25)	0.88 (38)
	2.5	1.54 (12)	0.73 (42)	0.49 (64)	0.52 (72)
	5	1.59 (28)	0.65 (67)	0.33 (87)	0.22 (94)
	10	2.05 (44)	0.75 (81)	0.63 (86)	0.46 (92)

proliferation

CI (% effect)		miR-130a-3p mimic (MOI)			
		0	2.5	5	10
miR-142-5p ASO (MOI)	0	1.91 (0)	0.81 (15)	0.90 (25)	1.40 (29)
	2.5	0.93 (13)	0.47 (47)	0.55 (54)	0.51 (73)
	5	0.88 (31)	0.46 (63)	0.21 (88)	0.27 (90)
	10	1.19 (42)	0.34 (84)	0.21 (93)	0.30 (92)

Supplementary Table 2. miR-142-5p and 130a-3p synergistically control Mφ profibrogenesis. Tables showing combination index (CI) values and % inhibition of collagen production and proliferation of co-cultured fibroblasts induced by IL-4 stimulated macrophages transduced with miR-142-5p ASO or/and miR-130a-3p mimics at various multiplicity of infection (MOI), compared to the untransduced macrophages. Reported CI values represent the average of four replicate experiments. CI values smaller than 0.5 indicate strong synergy.

Supplementary Table 3

Parameter	Sequence
**PCR primer**	
human GAPDH	5′-ATCACCATCTTCCAGGAGCGA-3′(forward)
	5′-CCTTCTCCATGGTGGTGAAGAC-3′(reverse)
human SOSC1	5′-CTGGTGCGCGACAGCCG-3′(forward)
	5′-ACGTAGTGCTCCAGCAGCTC-3′(reverse)
human PPAR-γ	5′-AATGACAGACCTCAGACAGATTG-3′(forward)
	5′-AAGGGAAATGTTGGCAGTGG-3′(reverse)
human HDAC1	5′-CATCTCCTCAGCATTGGCTT-3′(forward)
	5′-GACGGGGATGTTGGAAATTA-3′(reverse)
human HDAC2	5′-CATGCGGATTCTATGAGGCT-3′(forward)
	5′-ATGGCGTACAGTCAAGGAGG-3′(reverse)
human HDAC3	5′-TGTGTAACGCGAGCAGAACT-3′(forward)
	5′-GAGAGTCAGCCCCACCAATA-3′(reverse)
human HDAC4	5′-AGGATTCAGCAGCTCCACTG-3′(forward)
	5′-GAGCTCGTTGGAGCTATCGT-3′(reverse)
human HDAC5	5′-AACTGGGCATGGCTCTTG-3′(forward)
	5′-ACTCTCCCAACGAGTCGGAT-3′(reverse)
human HDAC6	5′-TCCAAGGCACATTGATGGTA-3′(forward)
	5′-CACAGTTCACCTTCGACCAG-3′(reverse)
human HDAC7	5′-CACTGGTGCTTCAGCATGAC-3′(forward)
	5′-GGCTCAGTCTTCCCCAGC-3′(reverse)
human HDAC8	5′-CCAGCACATAATCAGGACCA-3′(forward)
	5′-ATTTTGGGAGGAGGAGGCTA-3′(reverse)
human HDAC9	5′-CTTCTCACGGACAACAGGGT-3′(forward)
	5′-GCTCAGCAAAGAATGCACAG-3′(reverse)
human HDAC10	5′-GGCTGGAGTGGCTGCTATAC-3′(forward)
	5′-CTGAGGGAGGAGACAGAAGC-3′(reverse)
human HDAC11	5′-TATGGTTCCTCCTGTCTGGG-3′(forward)
	5′-GCACACGAGGCGCTATCTTA-3′(reverse)
mouse FIZZ1	5′-TGCTGGGATGACTGCTACTG-3′(forward)
	5′-AGCTGGGTTCTCCACCTCTT-3′(reverse)
mouse CCL17	5′-CAGGGATGCCATCGTGTTTC-3′(forward)
	5′- CACCAATCTGATGGCCTTCTT-3′(reverse)
mouse TGF-β1	5'- CCACCTGCAAGACCATCGAC -3′(forward)
	5'- CTGGCGAGCCTTAGTTTGGAC -3′(reverse)
mouse GAPDH	5′-GGCCTCACCCCATTTGATGT-3′(forward)
	5′-CATGTTCCAGTATGACTCCACTC-3′(reverse)

**Promoter and deletion constructs**	
miR142 (-1911/+91)	5’-CGGGGTACCCCCCACCTTAGGACCTGATC-3’ (forward)
miR142 (-1156/+91)	5’- CGGGGTACCCTCAGGTAAGGATCCGGACC-3’ (forward)
miR142 (-370/+91)	5’-CGGGGTACCCTGTGGCTGCCTCATTTGG-3’ (forward)
miR142 (-183/+91)	5’-CGGGGTACCGGAGGTAGAGGAGGCAAGTC-3’ (forward)
miR142 (-61/+91)	5’-CGGGGTACCCTCTTGGAGCAGG-3’ (forward)
	5’-CCGCTCGAGAGCCCACAGTACACTCATCC-3’ (reverse)
miR130a(-1891/+91)	5’-CGGGGTACCCAGTTGGGTGTTGAAGGAGA-3’ (forward)
miR130a(-1355/+91)	5’-CGGGGTACCCTTGCTGTGCAGCCTTAT-3’ (forward)
miR130a(-674/+91)	5’-CGGGGTACCTCTCAGTAACTGTGAGCGAATC-3’ (forward)
miR130a(-243/+91)	5’-CGGGGTACCGCTTTAGTGGGTCCTGTCC-3’ (forward)
	5’-CCGCTCGAGAGCACTACACGGCCAATG-3’ (reverse)
**Site-directed mutagenesis**	
STAT6 (TCTTAGGAA to CCTTAGGCC)	5’-GGGTTGGGGGGACCTTAGGCCGCCACAAGGAGGGC-3’ (forward)
SP-1 (CCCCTCCCC to CCACTCACC)	5’-GAGCCGGGGCCTCCACTCACCCATTTCCCCTCGG (forward)
**ChIP assay**	
STAT6 (180-nt product size)	5’- GGGGAAGGGAAGAGGGAAC-3’ (forward)<br>5’- CCTGACTCCTGCTCCAAGAG-3’ (reverse)
SP-1 (200-nt product size)	5’- GCCCCATCCCCTGCTGCT-3’ (forward)<br>5’- CAGGCCCAGCGACTCACC-3’ (reverse)
RNA pol II (103-nt product size)	5’- CAGGGACTGGGAGAAGGA-3’ (forward)<br>5’- CACTGCTAGTGACAGGTGCA-3’ (reverse)
AcH4, H3K4me3, H3K9me3 and H3K27me3,HDAC2 (200-nt product size)	5’- GCCCCATCCCCTGCTGCT-3’ (forward)<br>5’- CAGGCCCAGCGACTCACC-3’ (reverse)
**EMSA**	

STAT6	5’-TGGGGGGATCTTAGGAAGCCACAA-3’ (wild type)<br>5’-TGGGGGGACCTTAGGCCGCCACAA-3’ (mutant type)
SP-1	5’-CCGGGGCCTCCCCTCCCCCATTTCCT-3’ (wild type)<br>5’-CCGGGGCCTCCACTCACCCATTTCCT-3’ (mutant type)
Oct-1	5'-TGTCGAATGCAAATCACTAGAA-3'
**miRNA cloning in lentiviral vector**	
1.miR-142-5p ASO	5'- AGTAGTGCTTTCTACTTTATG- 3' (sense)
2.miR-142-5p ASO scramble nc	5'- GACCAAACATAGATGAACTTA- 3' (sense)
3.miR-130a-3p mimics	5'- CAGTGCAATGTTAAAAGGGCAT- 3' (sense)
4.miR-130a-3p mimics scramble nc	5'- GGTAACAATATCGGGTCAAGAT- 3' (sense)
**shRNA in lentiviral vector**	
shSOCS1-1	5'-GCACCTCCTACCTCTTCATGTTT- 3' (sense)
shSOCS1-2	5'-GACAATGCAGTCTCCACAGCA- 3' (sense)
shSTAT6-1	5'-ACAACACGTTGACTGATTCTT- 3' (sense)
shSTAT6-2	5'-AGCGGCTCTATGTCGACTTTC- 3' (sense)
shHDAC2-1	5’-GTACTACGCTGTCAACTTT-3’ (sense)
shHDAC2-2	5’-GACCGTCTCATTCCATAAA -3’(sense)
